# Tree regeneration trends in forests of the northeastern USA and their implications for resilience and restoration

**DOI:** 10.1002/eap.70288

**Published:** 2026-07-28

**Authors:** Lucas B. Harris, Melissa A. Pastore, Anthony W. D'Amato

**Affiliations:** ^1^ Rubenstein School of Environment and Natural Resources, University of Vermont Burlington Vermont USA; ^2^ USDA Forest Service, Northern Research Station, Forest Inventory & Analysis St. Paul Minnesota USA

**Keywords:** adaptation, climate change, forest inventory, forest resilience, northern temperate and boreal forest, restoration, tree regeneration

## Abstract

Tree regeneration is vital to shaping characteristics of future forests, including vulnerability to climate change; resilience to pests, pathogens and disturbance; and departure from historical species composition. We used data from nationwide forest inventory plots to investigate trends in sapling abundance and recruitment as well as tree density for common tree species from 2003 to 2023 across forests of the northeastern United States. Tree seedling composition was then examined using (1) predicted sapling recruitment based on seedling abundance and (2) abundance of small‐sized seedlings (5–15 cm and 15–30 cm tall). We assessed forest vulnerability to climate change using projected future habitat suitability, resilience using species‐level trait scores, and departure from historical composition based on early colonial land surveys, in all cases weighted by relative abundance of trees, saplings, and seedlings within forest inventory subplots. Sapling abundance and recruitment suggested improved climate change suitability relative to existing tree composition, yet also lowered resilience based on species trait scores. These seemingly contradictory results arise from declining sapling recruitment in northern tree species such as *Abies balsamea* and *Betula papyrifera* that are projected to fare poorly in future climates, yet persistently low sapling abundance of species that are more resilient to disturbances, pests, and pathogens, including *Acer rubrum*, *Acer saccharum* and *Quercus* species. Regeneration patterns suggested shifts away from historical species composition overall, although with considerable variability among genera. In contrast to saplings, composition of seedlings <30 cm tall indicated both decreased climate change vulnerability and greater resilience to various stressors and disturbance, suggesting that management targeted at survival and growth of already‐established seedlings could help to shape more resilient forests in the future.

## INTRODUCTION

The health of temperate and boreal forests is increasingly threatened by global change drivers, including climate change, more severe and widespread disturbance, and invasive pests and pathogens (Gauthier et al., 2015; Millar & Stephenson, 2015). Ongoing shifts in tree species composition affect how well forests will be able to withstand and adapt to these threats. Yet, forest trajectories are often challenging to predict given pervasive legacies of past land use and severe disturbance, which must be weighed against ongoing impacts of climate change, recent disturbance, forest pests, and forest management regimes (Duveneck et al., 2017). These complexities mean that empirical and process‐based models can arrive at notably different projections for future forest compositions (Iverson et al., 2017).

While composition of mature trees may be slow to respond to changing conditions, tree seedlings and saplings are generally more sensitive to environmental stressors and merit special attention as indicators of potential future tree composition (Grubb, 1977; Jackson et al., 2009). In forests of the eastern United States, tree regeneration is often sparse and compositionally dissimilar from canopy tree composition due to factors including overbrowsing by ungulates, invasive species, and climate change (Miller & McGill, 2019; Vickers et al., 2019). These same factors may increasingly threaten tree regeneration in European forests as well (Axer et al., 2021; Bödeker et al., 2023; Kuijper et al., 2010). Yet, the implications of shifting tree regeneration patterns in northern temperate and southern boreal forests are not well understood (Harris et al., 2024a; Knott et al., 2023).

Like many forested regions of the globe, modern forest composition in the northeastern United States has changed substantially over the past two centuries given the region's history of intensive harvesting and land clearing for agriculture followed by reestablishment of forests in the late 19th century to mid‐20th century (Ramankutty et al., 2010; Thompson et al., 2013). As forests continue to recover from this legacy of intensive land use that followed European colonization, tree regeneration patterns may increasingly reflect local environmental conditions, leading to a shift toward pre‐colonial composition. On the other hand, novel factors including heavy browsing pressure by deer, invasive pests and pathogens, and extreme weather events may push tree regeneration toward increasingly novel species composition (Miller & McGill, 2019). Current regeneration dynamics in the region may favor species compositions that are poorly suited to projected future climate (Noseworthy & Beckley, 2020; Zhu et al., 2014) and may also be less resilient to stressors such as drought and wildfire (Dey et al., 2019).

In the context of forest compositional shifts, trade‐offs among different aspects of forest resilience and restoration goals are likely to inform whether landscapes in this and other regions are best managed as historical, hybrid, or novel systems (Hobbs et al., 2014). As encapsulated in the resist‐accept‐direct (RAD) framework, managers face challenging decisions about where and when to resist ongoing anthropogenic changes by restoring historical species composition or maintaining current composition, accept changes with little to no intervention, or help to direct compositional shifts in ways that promote healthy and well‐functioning ecosystems (Schuurman et al., 2022). Three important considerations that factor into these decisions are (1) vulnerability to climate change; (2) resilience to pests, disease and disturbance; and (3) departure from historical composition. Our goal was to assess trends in tree composition and regeneration from 2003 to 2023 across forests of the northeastern United States and to consider how these trends inform each of the above considerations. Specifically, we set out to evaluate the implications of tree regeneration for (1) future change in climate suitability, (2) future change in forest resilience, and (3) restoration of historical composition as opposed to shifts toward novel species composition.

## METHODS

Patterns of tree seedling composition and abundances may provide the earliest indicators of future changes in forest composition, but may be highly uncertain due to high and variable mortality. Similarly, recruitment is an earlier indicator of potential change than total abundance, particularly given that trees may persist for decades at sapling and even seedling size in closed‐canopy forest (Canham, 1989; Marks & Gardescu, 1998). With this trade‐off between uncertainty and leading‐edge indicators in mind, we considered five metrics of tree species abundance across the northeastern United States (Table 1) derived from annual inventory data from the Forest Inventory and Analysis (FIA) program as described below.

**TABLE 1:** Metrics of tree species composition examined in this study.

Metric	Years (resolution)	*N* ^a^	Details
Tree density	2003–2023 (annual)	5654	Calculated from measurements of live trees ≥12.7 cm dbh
Sapling density	2003–2023 (annual)	5654	Stem density of live saplings 2.5–12.6 cm dbh
Sapling recruitment density	2008–2023 (annual)	5142	Observed density of recruitment between remeasurements, divided by the remeasurement period
Predicted sapling recruitment density	2024–2029 (measurement cycle)	2989	Predicted recruitment density from 2012 to 2023 Regeneration Indicator seedling abundance
Small seedling (5–15 cm and 15–30 cm tall) density	2012–2023 (measurement cycle)	2989	Stem density of the two smallest size classes in Regeneration Indicator protocols

^a^ Mean number of subplots per time period (year or measurement cycle).

### Trends in tree species abundance

Our first goal was to examine trends in annual time series of tree abundance, sapling abundance, and sapling recruitment across the northeastern United States. To do so, we leveraged tree measurements from the nationwide forest inventory conducted by the FIA program across Maine, New Hampshire, Vermont, Connecticut, Massachusetts, Rhode Island, and New York. The version of the FIA database (Gray et al., 2012) updated June 3, 2025 was used for analysis. The FIA plot design consists of four circular subplots (168 m^2^ each) within which trees (≥12.7 cm dbh) are sampled. Each subplot contains a microplot (13.5 m^2^) 3.7 m due east of the subplot center within which saplings (2.5–12.6 cm dbh) and seedlings (<2.5 cm dbh) are surveyed. For analysis, we selected all fully sampled subplots that lay entirely on accessible forest land as defined by FIA (Bechtold & Patterson, 2005) and which did not contain artificial regeneration. Although focusing on fully forested subplots is a commonly used approach, it excludes edge forests which differ from interior forests in ways that may affect regeneration, including through greater light availability and productivity rates (Morreale et al., 2021). Partially forested subplots in which the regeneration microplot was fully sampled made up 2.4% of all subplot measurements, and edge effects on tree regeneration in these subplots are a potential topic for future work. Standardized annual inventory protocols were initiated between 1999 and 2003 in the region, and plots were initially remeasured on a 5‐year cycle. Measurement cycles subsequently changed to 7 years for all states except Maine, which remained on the 5‐year cycle. Data were available through 2023 for all states at the time of analysis. Therefore, annual values were calculated from 2003 to 2023 for tree and sapling density (*n* = 118,737 subplots), and from 2008 to 2023 for sapling recruitment density (*n* = 82,277 remeasured annual inventory subplots).

Abundance was quantified using stem density by size class (Table 1) and then relativized at the subplot level for further analysis (e.g., the percentage of all live sapling stems in a subplot made up of a particular species). However, we also calculated trends in absolute abundance by species because absolute and relative abundance offer complementary information about forest demographics. Because measures of absolute abundance are affected by stand developmental stage (Oliver & Larson, 1996), we note that species‐level results should be interpreted in the context of both relative and absolute abundance. We investigated linear trends in annual means for common species for which live individuals (seedling, sapling, or tree size) were present in at least 100 subplots across all years of the analysis (*n* = 28, Appendix S1: Table S1). All trends were assessed using Sen's slope estimator (Sen, 1968), which is a nonparametric method that is robust to non‐normal distributions, and *p*‐values were adjusted for multiple comparisons within a given category (e.g., sapling abundance) using a Holm‐Bonferroni correction. Sen's slope estimator only evaluates linear trends, and we did not test for nonlinear annual trends. Species‐level trends in the tree layer were similar using basal area and density, suggesting that the use of density as opposed to basal area to represent tree composition did not strongly influence results (Appendix S1: Table S2).

For species and metrics with significant trends, maps were generated showing abundance in the first and last year analyzed. The three highest magnitude trends for relative sapling abundance and absolute sapling recruitment are shown here (see Appendix S1: Figures S1–S13 for maps of all other significant trends). For visualization purposes, abundance was interpolated to 5‐km grid squares using inverse distance weighted interpolation considering the 200 nearest subplots and an inverse distance power of 0.01 via the “gstat” R package (Gräler et al., 2016).

### Seedling composition

Tree seedling composition (<2.5 cm dbh) was assessed using Regeneration Indicator (RI) plots, which are a subset of all FIA plots across the northern United States (1/8 of plots within our study region) in which seedlings are tallied by six height classes for both hardwoods and softwoods instead of one size class for hardwoods (≥30 cm tall) and one size class for softwoods (≥15 cm tall) (McWilliams et al., 2015). The more detailed RI seedling surveys, which were initiated in 2012, allow for assessments of factors limiting seedling survival and improved predictions of potential future forest structure and composition (Harris et al., 2024b; Vickers et al., 2019). We did not assess annual trends using RI but rather grouped the initial cycle of RI measurements (Time 1, 2012–2018) and the second cycle of RI remeasurements (Time 2, 2018–2023) for two reasons: the smaller sample size of the RI dataset (*n* = 5978 subplot measurements), which we considered insufficient for capturing annual trends; and to account for high interannual variability in the abundance of small‐sized seedlings due to mast years and other factors. Similarly, RI measurements from a single year were considered too noisy and uncertain to accurately predict sapling recruitment (see following paragraph).

We calculated two seedling composition metrics. First, we considered stem density within the two smallest RI seedling size classes (established seedlings 5–15 cm tall and 15–30 cm tall). Abundance within these smallest classes approximates the results of seedling establishment and early survival, prior to most compositional shifts driven by differential seedling mortality. Second, we used stem density within the RI height classes in Time 1 as well as other stand and site‐level covariates to generate statistical models predicting the presence of sapling recruitment by species in Time 2 (Harris et al., 2022, 2024b) (see Appendix S2). To calculate expected sapling recruitment density, the predicted likelihood of recruitment for each species was calculated from each subplot measurement (Time 1 and Time 2) and multiplied by the mean density of sapling recruits where present (see Appendix S2).

### Future climate suitability

To assess stand‐level vulnerability to climate change, we used species‐level predictions of current and future habitat suitability from the Climate Change Tree Atlas (Iverson et al., 2019). These models predict the current and projected future importance value (a measure of relative abundance) of individual tree species within either 10‐ or 20‐km grid squares, which indicates future climate risk (Iverson et al., 2019). We calculated the ratio of future to present predicted importance values for each species within each grid square, using end‐of‐century projections under the 8.5 Representative Concentration Pathway (Iverson et al., 2019). These ratios were converted into five change classes in suitable habitat (large decrease, decrease, no change, increase, and large increase) following the criteria of Janowiak et al. (2017), again for each species and grid square. For example, a common tree species forecast to decline by >50% by the end of the century in a given 10‐km grid square was assigned “large decrease,” whereas a common species forecast to more than double in abundance was assigned “large increase” (Janowiak et al., 2017). Then, we used relative species abundance of the five species composition metrics (Table 1) to calculate the percentage of each subplot falling within each change class, that is, the percentage of stems forecast to experience large decreases, decreases, little to no change, increases, or large increases in abundance based on end‐of‐century climate suitability. These subplot‐level percentages were subsequently averaged by year or measurement cycle to assess temporal trends.

### Forest resilience based on species traits

To assess potential changes in forest resilience based on species traits, we used species‐level scores representing biological (e.g., shade tolerance, edaphic specificity, dispersal) and disturbance‐related (e.g., insect pests, disease, drought, fire) characteristics that Matthews et al. (2011) compiled from a literature review of species life‐history traits and scaled between −3 (least resilient) and +3 (most resilient). These characteristics are indicators of resilience, or the capacity of a system to withstand stressors and recover (Gunderson, 2000). Scores were generated for each FIA subplot based on relative abundance of the five species composition metrics (Table 1) and were aggregated by year or measurement cycle to assess trends.

### Departure from precolonial composition

Descriptions of “witness trees” from early colonial land surveys have been used to reconstruct forest composition prior to European colonization and to compare precolonial and modern composition (Cogbill et al., 2002; Thompson et al., 2013). We compared relative abundance of witness trees by genus compiled at the town level across the northeastern United States (Thompson et al., 2013; Thompson & Cogbill, 2023) with relative abundance metrics that we derived from FIA data (Table 1).

To facilitate comparison with witness tree records, we limited this portion of the analysis to subplot measurements within the 423 towns in New England and New York that Thompson et al. (2013) identified as having sufficient witness tree and FIA records to compare precolonial with modern tree composition. This subset of towns included 30,724 FIA subplot measurements, 20,758 remeasurements assessed for sapling recruitment, and 1430 RI measurements.

Witness tree records were compiled by groups of species, mainly by genera, to accommodate the fact that trees were often not identified at the species level (Cogbill et al., 2002). We selected the 13 groups from the witness tree dataset that were present in each year of the FIA data to compare modern with precolonial tree composition. Finally, we assessed mean departure from precolonial composition for each year or measurement cycle using robust Aitchison's distance, which is designed for use with compositional data and is considered superior to other distance measures when considering proportional abundance (Aitchison, 1982; Martino et al., 2019).

## RESULTS

### Species‐level trends

**FIGURE 1:**
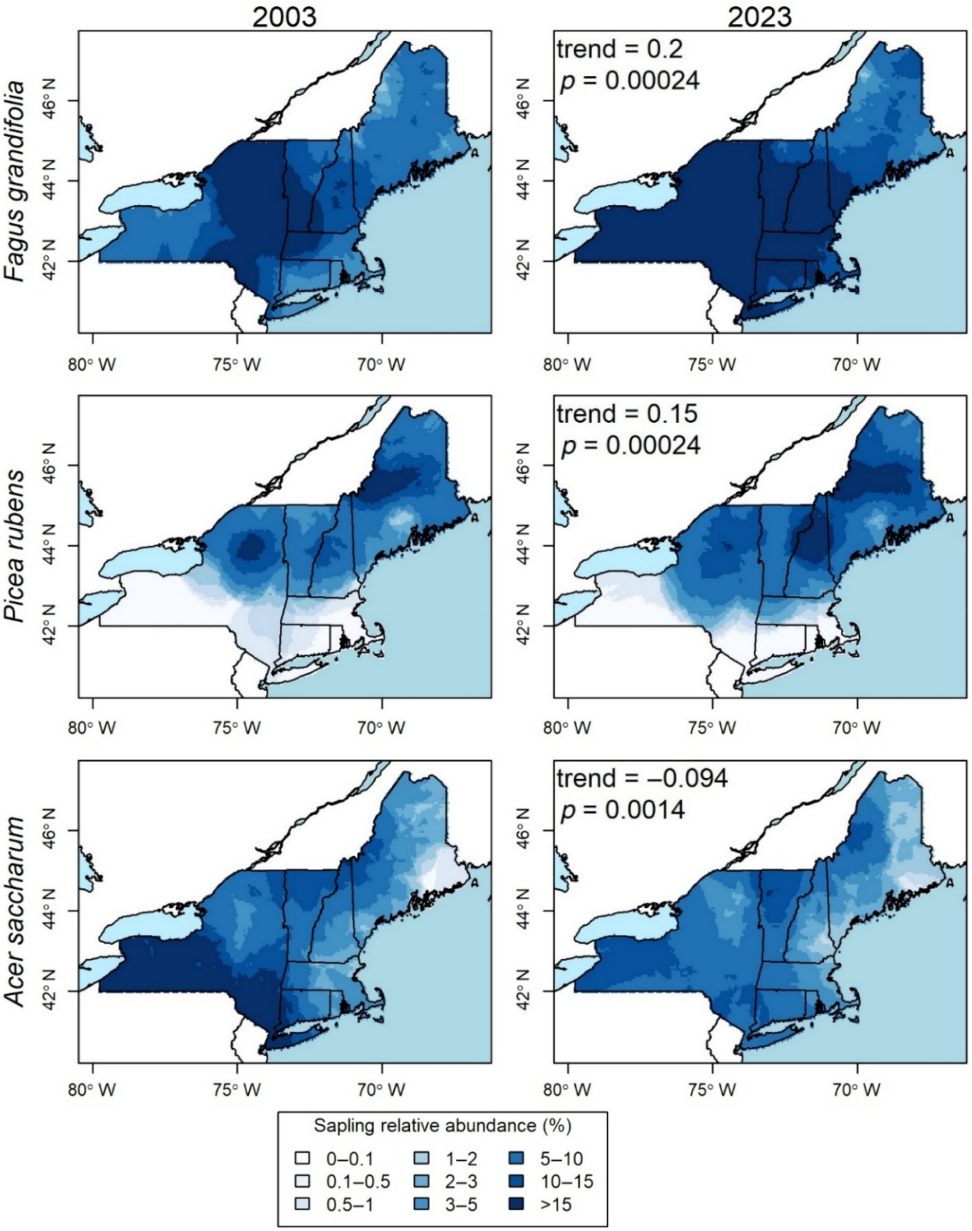
Interpolated maps of relative sapling abundance based on stem density at the start and end years of the analysis (2003 and 2023) for the three species with the highest magnitude linear trends: *Fagus grandifolia* (beech), *Picea rubens* (red spruce), and *Acer saccharum* (sugar maple). Estimated linear annual trends and *p*‐values from Sen's slope tests are shown at the upper left of the year 2023 panels.

**FIGURE 2:**
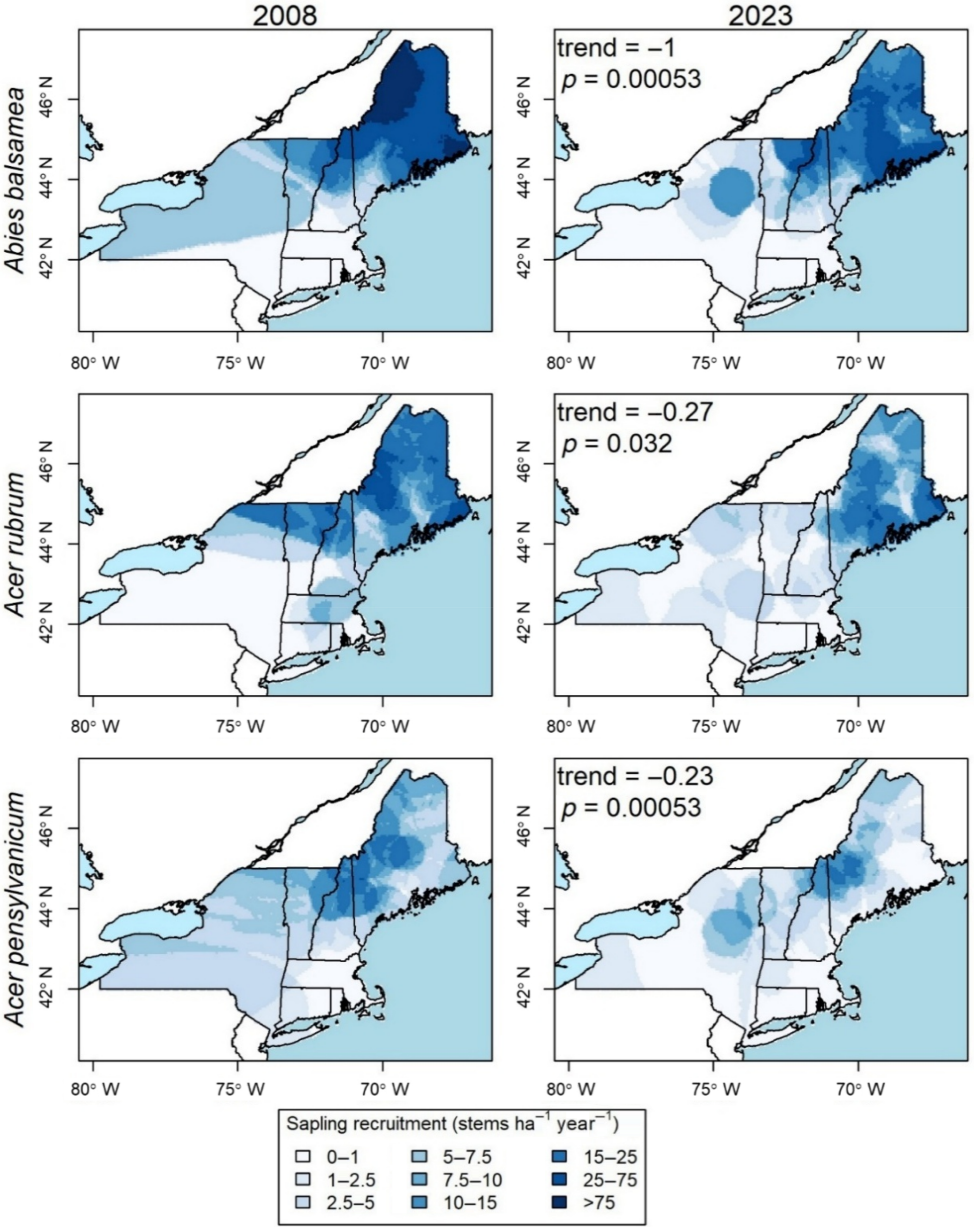
Interpolated maps of sapling recruitment rates at the start and end years of the analysis (2008 and 2023) for the three species with the highest magnitude linear trends: *Abies balsamea* (balsam fir), *Acer rubrum* (red maple), and *Acer pensylvanicum* (striped maple). Estimated linear annual trends and *p*‐values from Sen's slope tests are shown at the upper left of the year 2023 panels.

From 2003 to 2023, *Abies balsamea*, *Acer pensylvanicum*, *Betula alleghaniensis*, and *Fagus grandifolia* increased in both absolute and relative live tree density (Table 2). Absolute tree density also increased for *Picea rubens*. Relative tree density decreased for *Acer saccharum*, *Betula papyrifera*, *Populus tremuloides*, and *Quercus alba*. In the sapling layer, three species increased in both absolute and relative terms (*Pic. rubens*, *Fa. grandifolia*, and *Thuja occidentalis*) and six species decreased in both terms, most notably *Ac. saccharum*, *B. papyrifera*, and *Prunus serotina*. In addition, *Pinus strobus*, *Quercus rubra*, and *Betula populifolia* decreased in relative, although not absolute, sapling abundance (Table 2). Relative increases in *Fa. grandifolia* saplings were evident throughout the region, while increases in *Pic. rubens* were concentrated in northern and mountainous areas, and decreases in *Ac. saccharum* were concentrated in western and southern New York (Figure 1). Absolute sapling recruitment decreased for six species (*Ab. balsamea*, *Ac. pensylvanicum*, *Acer rubrum*, *Ac. saccharum*, *B. papyrifera*, and *Pr. serotina*), whereas relative recruitment increased for *Fa. grandifolia* and *Pic. rubens*. For the three species that showed the greatest declines in recruitment rates (*Ab. balsamea*, *Ac. rubrum*, and *Ac. pensylvanicum*), these declines occurred largely in northern Maine into northern New Hampshire and Vermont (Figure 2). Interestingly, comparison of small seedling (5–15 cm tall and 15–30 cm tall) absolute abundance at Time 1 and Time 2 suggests that changes in small seedling abundance did not track changes in saplings or sapling recruits (Appendix S1: Table S3). For example, absolute abundance of 5–15‐cm‐tall seedlings was actually greater at Time 2 than Time 1 for *Ac. pensylvanicum* and *Ac. saccharum*, despite the declines in absolute sapling recruitment observed for these species (Appendix S1: Table S3). Indeed, *Ac. rubrum* and *Ac. saccharum* had the highest relative abundance among both trees and 5–15‐cm‐tall seedlings in the 2018–2023 measurement cycle, but were proportionally less abundant among saplings and sapling recruits (Appendix S1: Table S4).

**TABLE 2:** Linear trends in absolute and relative abundance of trees (≥12.7 cm dbh), saplings (2.5–12.6 cm dbh), and sapling recruitment indicated by Sen's slope with Holm‐corrected *p*‐values.

Species	Tree density (stems ha^−1^ year^−1^)		Sapling density (stems ha^−1^ year^−1^)		Recruitment (stems ha^−1^ year^−1^)	
	Absolute	Relative (%)	Absolute	Relative (%)	Absolute	Relative (%)
*Abies balsamea*	**1.8 (<0.001)**	**0.18 (0.004)**	2.2 (1)	0.12 (0.384)	**−1.0 (<0.001)**	**−0.53 (0.036)**
*Acer pensylvanicum*	**0.043 (0.002)**	**0.0085 (0.006)**	0.27 (1)	0.0037 (1)	**−0.23 (<0.001)**	−0.08 (1)
*Acer rubrum*	−0.0068 (1)	−0.044 (0.8)	0.012 (1)	−0.065 (0.108)	**−0.27 (0.032)**	−0.061 (1)
*Acer saccharum*	−0.18 (1)	**−0.073 (0.048)**	**−1.1 (0.046)**	**−0.094 (0.001)**	**−0.088 (0.003)**	−0.057 (1)
*Acer spicatum*	0 (1)	0 (1)	**−0.58 (0.004)**	**−0.031 (0.002)**	−0.037 (0.206)	−0.037 (0.498)
*Betula alleghaniensis*	**0.47 (<0.001)**	**0.083 (<0.001)**	0.24 (1)	0.022 (1)	−0.058 (1)	0.028 (1)
*Betula papyrifera*	−0.049 (1)	**−0.035 (0.027)**	**−1.1 (0.003)**	**−0.029 (0.036)**	**−0.12 (0.013)**	−0.075 (0.086)
*Betula populifolia*	0.021 (0.423)	0.0021 (1)	−0.32 (0.517)	**−0.019 (0.048)**	−0.029 (0.793)	0.0011 (1)
*Fagus grandifolia*	**0.4 (<0.001)**	**0.075 (0.01)**	**3.6 (<0.001)**	**0.2 (<0.001)**	−0.078 (1)	**0.39 (0.027)**
*Picea rubens*	**0.84 (<0.001)**	0.076 (0.376)	**4.5 (<0.001)**	**0.15 (<0.001)**	−0.2 (0.323)	**0.23 (0.021)**
*Pinus strobus*	−0.21 (0.091)	−0.046 (0.056)	−0.52 (0.273)	**−0.049 (<0.001)**	−0.11 (0.075)	−0.026 (1)
*Populus tremuloides*	−0.016 (1)	**−0.022 (0.002)**	−0.73 (0.053)	−0.026 (0.08)	0.033 (1)	0.034 (1)
*Prunus pensylvanica*	−0.0073 (1)	−0.0051 (0.108)	**−0.52 (0.003)**	**−0.017 (0.002)**	−0.047 (0.684)	−0.013 (1)
*Prunus serotina*	−0.078 (0.195)	−0.024 (0.078)	**−0.64 (<0.001)**	**−0.046 (<0.001)**	**−0.055 (0.005)**	−0.051 (0.145)
*Quercus alba*	**−0.063 (0.035)**	**−0.019 (0.023)**	−0.048 (0.273)	−0.0071 (0.474)	−0.0057 (0.323)	−0.011 (0.498)
*Quercus rubra*	−0.056 (1)	−0.0081 (1)	−0.15 (0.09)	**−0.018 (0.036)**	−0.008 (1)	0.011 (1)
*Thuja occidentalis*	0.075 (1)	−0.0057 (1)	**1.2 (0.009)**	**0.037 (0.021)**	0.0018 (1)	0.063 (1)
*Ulmus americana*	−0.037 (0.632)	−0.016 (0.122)	**−0.23 (0.017)**	**−0.023 (0.03)**	−0.003 (1)	0.0072 (1)

*Note*: Significant trends (*p* < 0.05) are in bold.

### Future climate suitability

**FIGURE 3:**
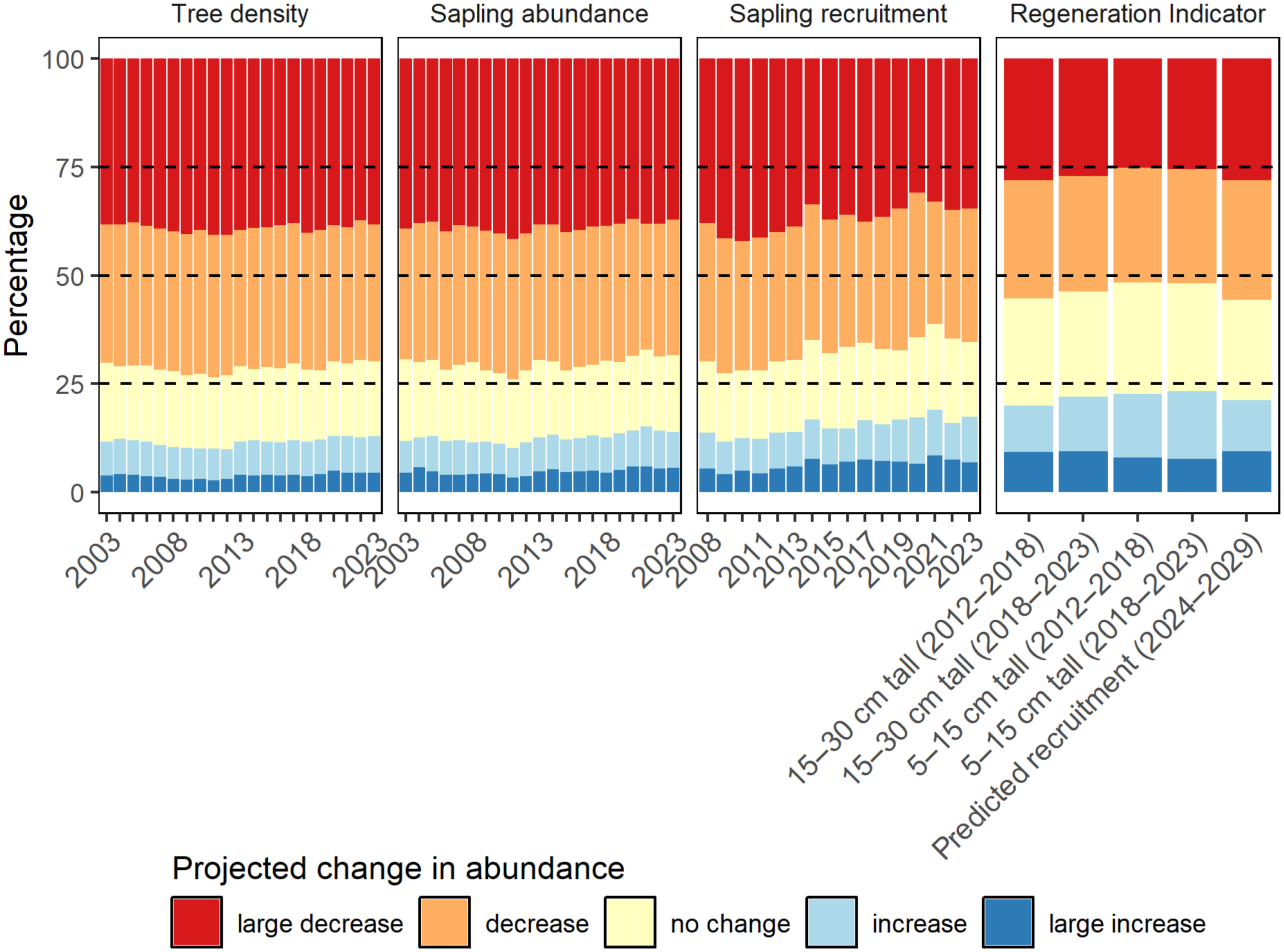
Percentage abundance of trees, saplings, and seedlings within different projected climate change classes, which represent the direction and magnitude of expected change in habitat suitability as quantified by the ratios between current and projected end‐of‐century species importance from the Climate Change Tree Atlas (CCTA) (Janowiak et al., 2017). These percentages were calculated by matching forest inventory subplot measurements with corresponding projections within 10‐ or 20‐km grid cells used by the CCTA (Peters et al., 2019a, 2019b) and then averaging results across the study area. Annual values are shown for relative abundance of trees, saplings, and sapling recruits with estimated linear trends shown in Appendix S1: Table S5. Abundance of seedlings 5–15 cm tall and 15–30 cm tall from Regeneration Indicator subplots are also shown by time period (2012–2018 and 2018–2023) along with predicted sapling recruitment (2024–2029).

The analysis of future climate suitability, which summarizes the direction and magnitude of expected change in habitat suitability by the end of the century assessed by tree species and location, showed that >60% of trees, saplings, and sapling recruits fell into the “large decrease” or “decrease” categories across all years of the annual inventory (Figure 3) indicating a mismatch between current species composition and projected future climate suitability. However, the percentage of saplings in the “increase” category had a significant positive trend as did the percentage of sapling recruits in the “increase” and “large increase” categories (Figure 3, Appendix S1: Table S5). A negative trend was also observed for sapling recruits in the “large decrease” category. Predicted sapling recruitment suggests that these trends will continue in the near future. Climate risk was lower overall for small seedlings (15–30 cm tall and especially 5–15 cm tall) than for trees, saplings, or sapling recruits (Figure 3).

### Resilience based on species traits

**FIGURE 4:**
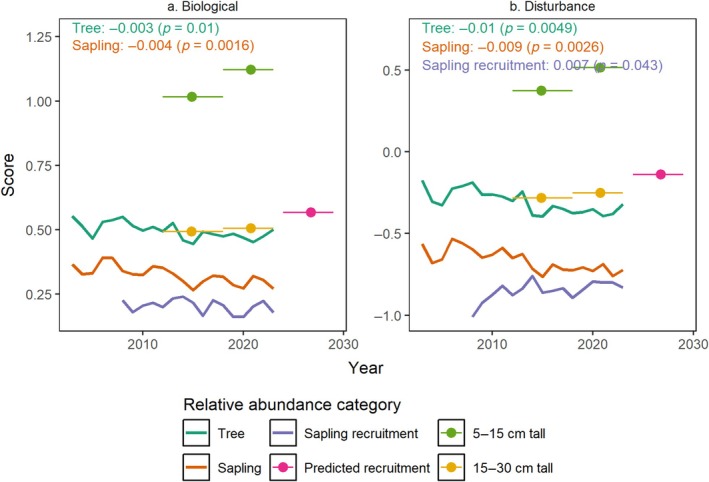
Forest resilience as indicated by mean (a) biological and (b) disturbance scores based on metrics of tree, sapling, and seedling relative abundance. Species‐level scores range from −3 (low resilience) to +3 (high resilience) and indicate (a) ability to tolerate and recover from an array of abiotic and biotic stressors; and (b) ability of species to tolerate specific disturbances including disease, insects, drought, wind, and harvesting (Matthews et al., 2011). Significant linear trends in mean annual scores (*p* < 0.05, Sen's slope test) are shown with corrected *p*‐values at the upper left of each panel. Abundance of small seedlings 5–15 cm and 15–30 cm tall as well as near‐term predictions of sapling recruitment from Regeneration Indicator subplots are averaged by time period with the mean (points) and range (line) of each time period shown.

According to species‐level biological and disturbance trait scores, seedlings 5–15 cm tall had the highest resilience across all years, followed by seedlings 15–30 cm tall, then trees, saplings, and finally sapling recruits (Figure 4). Resilience, as indicated by both biological and disturbance trait scores, decreased significantly over time for trees and saplings (Figure 4). However, disturbance trait scores increased over time for sapling recruits (Figure 4b) and predicted sapling recruitment suggested ongoing increases in disturbance trait scores.

### Departure from precolonial composition

**FIGURE 5:**
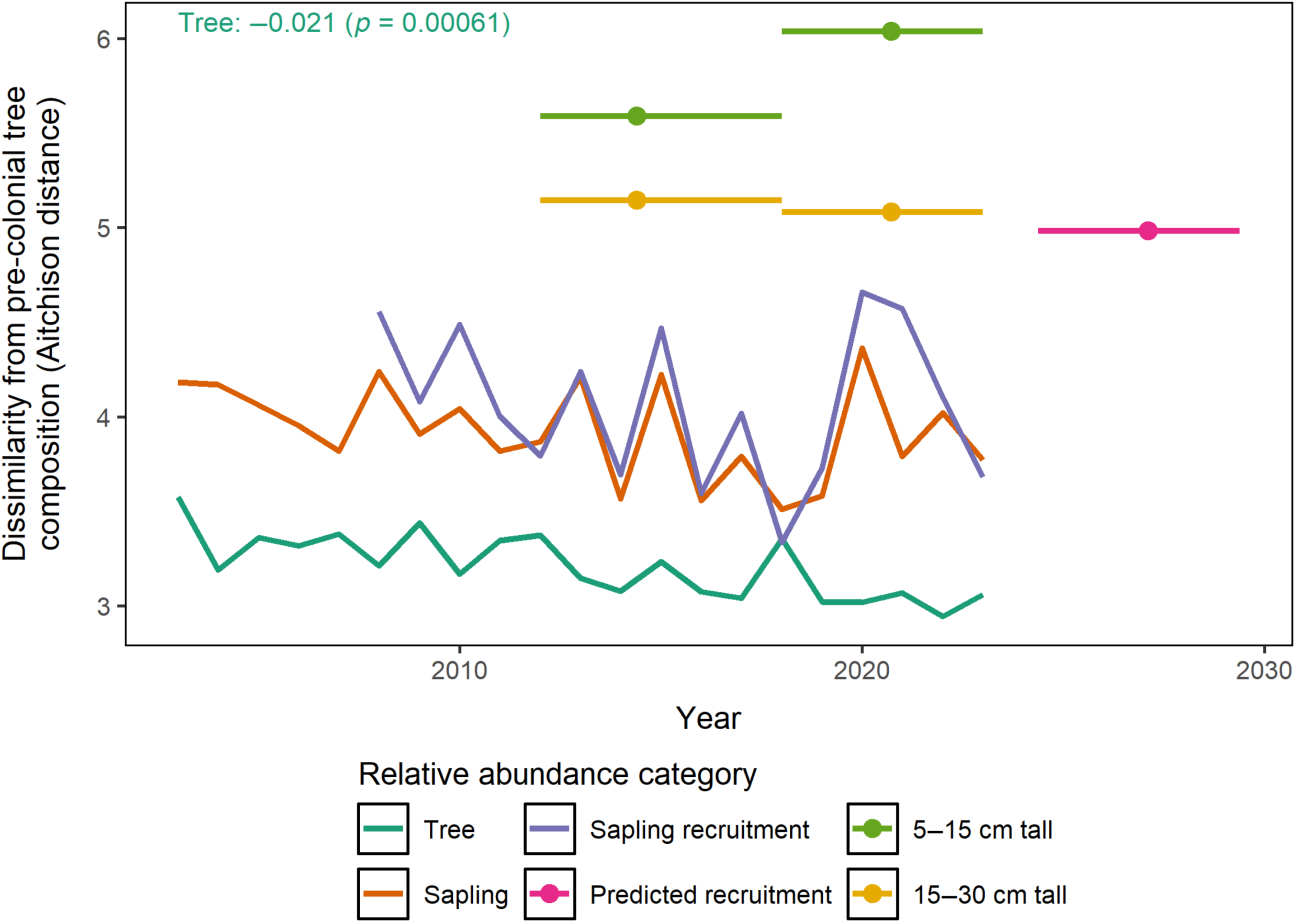
Annual mean dissimilarity from precolonial tree composition (robust Aitchison distance) for relative abundance of trees, saplings, and seedlings. *Y*‐axis values indicate the degree of departure from forest composition prior to European colonization as assessed using witness tree records from early colonial land surveys (Thompson et al., 2013). Dissimilarity of trees, saplings, and sapling recruits is averaged by year, and estimates of linear annual trend and corrected *p*‐value from Sen's slope test are shown for the one significant trend (in tree dissimilarity) at the upper left. Abundance of small seedlings 5–15 cm and 15–30 cm tall, as well as near‐term predictions of sapling recruitment from Regeneration Indicator subplots, are averaged by time period with the mean (points) and range (line) of each time period shown.

**FIGURE 6:**
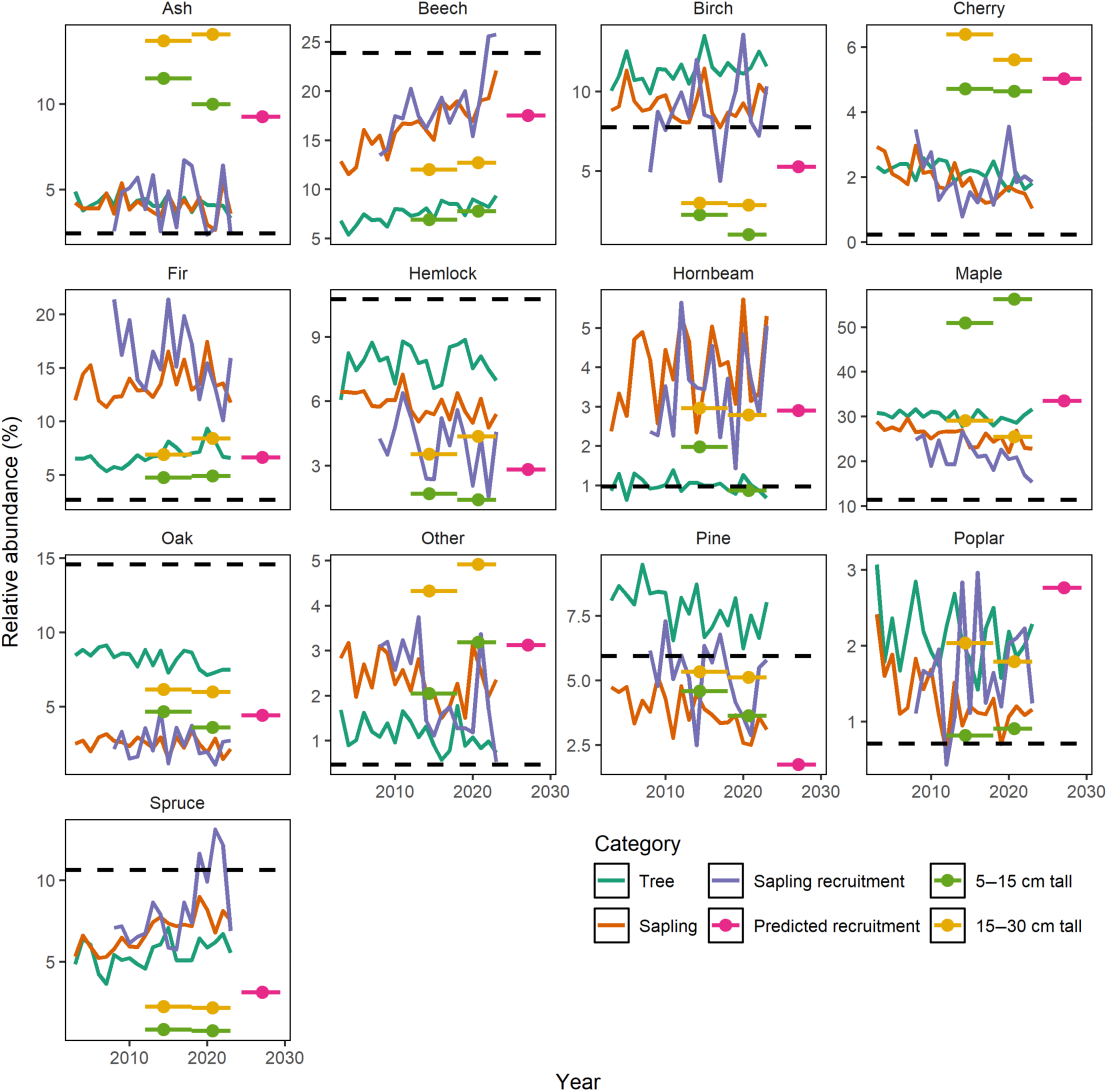
Relative abundance by tree species genus/group compared with precolonial tree composition estimated from early colonial land surveys from 423 towns in the northeastern United States (dashed lines). Annual means are given for trees, saplings, and sapling recruits. Abundance of small seedlings 5–15 cm and 15–30 cm tall as well as near‐term predictions of sapling recruitment from Regeneration Indicator subplots are averaged by time period with the mean (points) and range (line) of each time period shown. Note that *y*‐axis limits vary among panels.

Dissimilarity from precolonial composition decreased significantly over time for trees (*p* < 0.001), although tree composition was already closer to precolonial forest composition than saplings or sapling recruits (Figure 5). Composition of small seedlings was the least similar to precolonial composition. A genus‐level comparison (Figure 6) shows the complexity underlying the overall composition scores. Regeneration patterns appeared to be forecasting a return toward precolonial composition for spruce (*Picea*), beech (*Fagus*), pine (*Pinus*), birch (*Betula*), and maple (*Acer*), although it should be noted that the latter was heavily represented among small seedlings (Figure 6). Meanwhile, tree regeneration suggested future overrepresentation of fir (*Abies*) and hornbeam (which includes *Ostrya* and *Carpinus*). Finally, hemlock (*Tsuga*) and oak (*Quercus*) were already underrepresented in the tree layer and further underrepresented among saplings and sapling recruits. Sapling abundance within the towns used for the witness tree comparison declined significantly for maple and cherry (*Prunus*) and increased significantly for spruce and beech, all of which was consistent with a return toward precolonial composition (Appendix S1: Table S6).

### Synthesis

**FIGURE 7:**
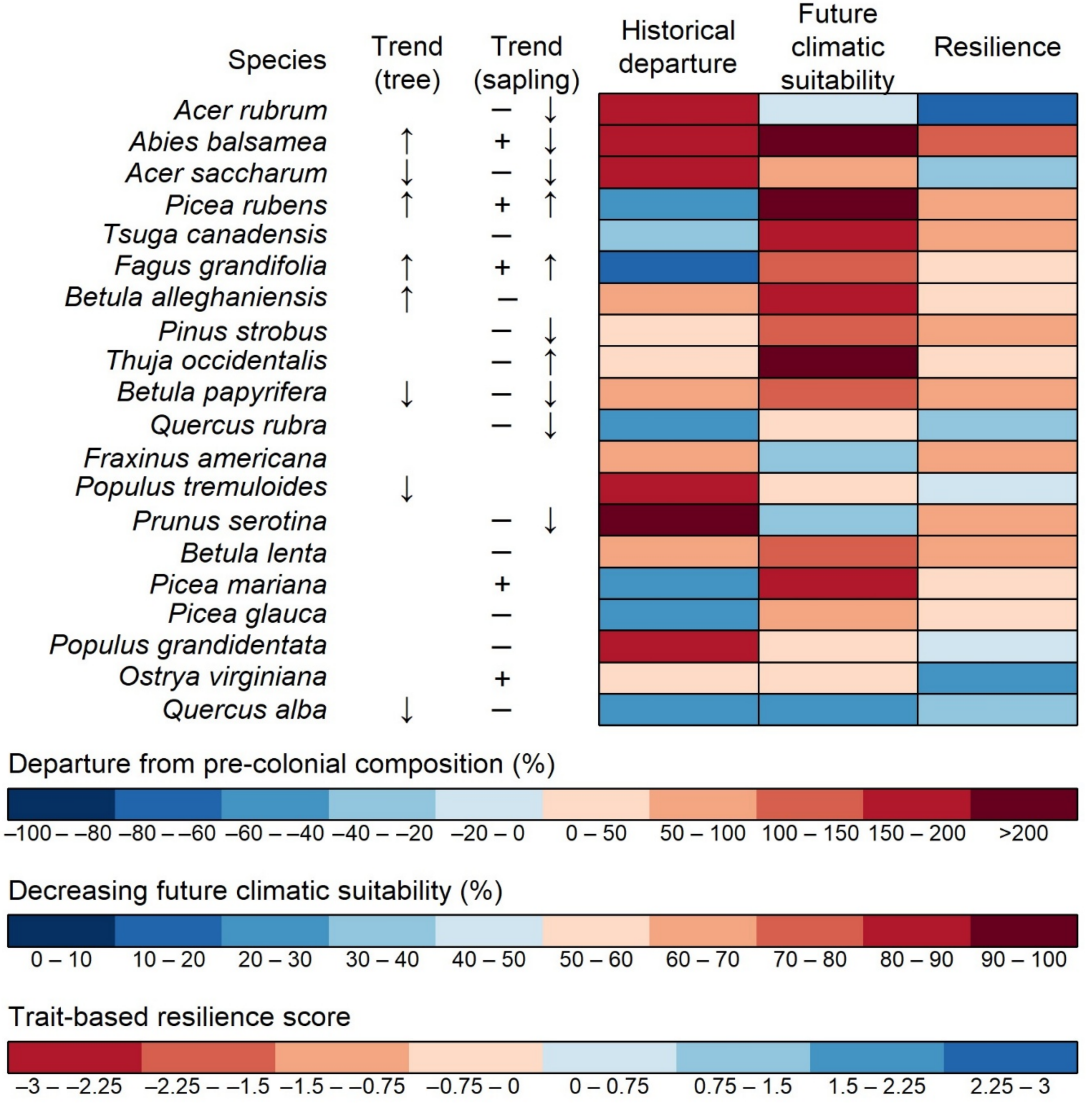
Summary figure illustrating species‐level trends and tradeoffs between restoration, climate vulnerability and resilience to pests and disturbances. In each case, blues (reds) indicate that increases (decrease) in a species would be beneficial. The 20 most abundant species by live tree density from the most recent measurement cycle (2018–2023, most abundant at top) are shown. Arrows indicate significant positive or negative trends (*p* < 0.05, Sen's slope test) and “+” (“−”) indicates greater (lesser) relative abundance among saplings and recruited saplings compared with trees. Historical departure and climatic suitability were calculated from forest inventory subplots in the most recent measurement cycle, weighted by tree density. Note that historical departure was assessed at the genus level but is noted beside each species here. Biological and disturbance scores from ModFacs (Matthews et al., 2011) were averaged to produce a trait‐based resilience score for each species.

Summarizing results at the species level (Figure 7) illustrates both synergies and tradeoffs between climate risk, resilience, and restoration. Oak (*Quercus*) species had the greatest synergy as oaks are underrepresented compared to the precolonial period, underrepresented and in decline in the regeneration layer, and score highly in climatic suitability and trait‐based resilience (Figure 7). On the other end of the spectrum, *Ab. balsamea* was overrepresented compared to the precolonial period and had low climatic suitability and low resilience scores. Another group of species including maples (*Acer*) and aspens (*Populus*) was more abundant compared with the precolonial period and in decline in the regeneration layer, yet had relatively favorable climatic suitability and resilience scores. Finally, spruce (*Picea*) was returning closer to its precolonial abundance yet had low climatic suitability scores.

## DISCUSSION

Like other temperate and southern boreal forests globally, forest trajectories in the northeastern United States are being shaped both by ongoing recovery from intensive land use and by novel influences, including increased herbivory, invasive species, altered disturbance regimes, and climate change (Côté et al., 2004; Duveneck et al., 2017; Fei et al., 2019). These complex and countervailing influences have generated debate about the extent to which active as opposed to passive management approaches promote ecological integrity (Faison, Laflower, et al., 2023; Faison, Masino, & Moomaw, 2023). Assessments of tree regeneration trends as presented here can provide valuable information about forest trajectories to inform management strategies (Bose et al., 2017a). In particular, our assessment of sapling recruitment serves as a leading‐edge indicator of change in the sapling layer. By examining small‐sized seedlings (5–15 cm and 15–30 cm tall), we also can separate composition arising from fecundity and seedling establishment, which are key processes in tree demography (Sharma et al., 2022), from subsequent environmental filtering that may dramatically alter composition along the path to sapling recruitment. Indeed, the strong differences between composition of 5–15 cm and 15–30 cm tall seedlings that we observed are consistent with prior work suggesting that factors such as herbivory and shrub cover generate differences in survival between these two height classes both within and among species (Harris et al., 2024b, 2025).

By investigating both canopy tree and regeneration trends, we were able to identify species undergoing sustained changes in abundance (e.g., increases in American beech [*Fa. grandifolia*] and red spruce [*Pic. rubens*] and decreases in sugar maple [*Ac. saccharum*]), species that are stable in the tree layer but may experience future declines based on regeneration, and species that may be reaching an inflection point as they continue to increase in the canopy even as sapling abundance or recruitment declines (e.g., balsam fir [*Ab. balsamea*]). We found that trends in regeneration, as compared with those of canopy trees, suggest slightly lower vulnerability to climate change (based on future habitat suitability) yet increased vulnerability to disturbances, pests, and pathogens (based on species‐level traits). Meanwhile, composition of small‐sized seedlings (15–30 cm tall and especially 5–15 cm tall) was more favorable than tree or sapling composition from both a climate vulnerability and trait‐based resilience perspective. We also found that canopy tree composition has become more similar to precolonial composition over time, yet regeneration patterns suggest increased novelty. Below, we interpret our results in the context of prior work and discuss implications for management strategies.

### Future climate suitability

Our analysis of future climate suitability showed a mismatch between current species composition and projected changes in habitat suitability that has been noted by others (e.g., Noseworthy & Beckley, 2020), with all size classes (trees, saplings, sapling recruits and small seedlings) indicating future declines in habitat suitability on average. However, we also found that sapling recruits were less vulnerable to projected end‐of‐century climate change than saplings or trees and that small seedlings were less vulnerable still. These patterns suggest that the mismatch between current composition and projected habitat suitability could lessen in the near future, particularly with management to promote less vulnerable species already present in the small seedling layer. Because trees can tolerate a broader range of environmental conditions than seedlings or saplings, our findings could reflect ongoing impacts of climate change under which trees are persisting in areas no longer climatically suitable for regeneration of a particular species (Jackson et al., 2009). For example, declining balsam fir (*Ab. balsamea*) sapling recruitment and its low relative abundance in the seedling layer are likely key contributors to the overall lower climate vulnerability of sapling recruits and seedlings in our analysis.

### Forest resilience

We evaluated forest resilience based on species‐level trait scores indicating biological characteristics that confer resilience to a range of biotic and abiotic conditions as well as the ability of species to resist and recover from disturbance (Matthews et al., 2011). In both cases, the sapling layer was less resilient than the tree layer, yet the small seedling layer had the most resilient composition. These results indicate that environmental filtering between the time of seedling establishment and sapling recruitment is leading to less resilient forest composition, with implications for how forests in the northeastern United States will be able to respond to likely future increases in natural disturbance such as windstorms, droughts, fires, and spread of nonindigenous insects and pathogens (Anderegg et al., 2022; Fei et al., 2019).

We offer three potential explanations for the less resilient composition of saplings than trees or small seedlings: (1) Decreased extent and intensity of disturbance (including harvesting) since the late 1800s to mid‐1900s following intense harvests or abandonment of agricultural land have allowed stands to mature and accumulate biomass on average in eastern United States forests (Canham et al., 2024). These changes favor shade‐tolerant, late successional species that are less tolerant of disturbance. (2) A decrease in fire activity since European colonization has contributed to increases in mesophytic, shade‐tolerant species at the expense of disturbance‐adapted species (Knott et al., 2019; Nowacki & Abrams, 2008; Roos, 2020; Tulowiecki et al., 2025). (3) High and increasing browsing by white‐tailed deer (*Odocoileus virginiana*) may create a regeneration bottleneck for key resilient species, including oaks (*Quercus*), red maple (*Ac. rubrum*), and sugar maple (*Ac. saccharum*). This can create a population structure with abundant small‐sized seedlings yet sparse saplings, which is consistent with our results (Harris et al., 2025; Henry & Walters, 2023; Lesser et al., 2019; Miller et al., 2023).

### Departure from precolonial forest composition

In the northeastern United States, precolonial forest composition inferred from witness tree records can serve as an important baseline for assessing the novelty of current forest composition and informing restoration goals (Cogbill et al., 2002; Thompson et al., 2013). Interestingly, we found that tree composition was slowly returning toward precolonial composition over the past two decades, whereas regeneration favored increased novelty. Given that old forests largely dominated by shade‐tolerant species were predominant prior to European colonization (Lorimer & White, 2003), forest maturation should tend to restore historical tree composition, as we observed, so long as this is not outweighed by novel influences on regeneration and tree mortality. The greatest contributor to increased sapling novelty, according to our analysis, was underrepresentation of oak saplings. Oak regeneration is low in eastern United States forests due to factors including declining fire activity and browsing by deer (Dey, 2014; Woodall et al., 2008). Despite widely noted increases in beech sapling abundance since the 1980s in the northeastern United States, driven in part by beech bark disease (Bose et al., 2017a, 2017b), our results indicate that beech sapling abundance is only now approaching the historical abundance of beech in the tree layer. However, the emergence of beech leaf disease, which was discovered in the midwestern United States in 2012, poses new and serious threats to seedlings and saplings, with the potential to dramatically affect regeneration dynamics in the region and drive further shifts toward increased novelty (Reed et al., 2022).

### Management implications

Regeneration is an increasing focus of forest management, including artificial regeneration efforts that globally include initiatives such as One Trillion Trees (https://www.1t.org/) and within the northeastern United States includes New York's “25 Million Trees by 2033” (https://dec.ny.gov/nature/forests-trees/climate-change/25-million-trees). Silvicultural practices that encourage natural regeneration can serve as a valuable and cost‐effective complement to artificial regeneration, particularly when species recruited support desired ecological, cultural, and social values and are resilient to changing climate and disturbance regimes. We found that the composition of small‐sized seedlings was less similar to precolonial composition than current canopy tree composition, yet may be more resilient to likely future conditions. Therefore, strategically improving survival and recruitment of existing natural regeneration could help to accomplish climate adaptation goals. Many priority species, including northern red oak (*Q. rubra*) and sugar maple, appeared to have sufficient and stable abundance of naturally regenerated small‐sized seedlings at a regional scale yet were experiencing declines in sapling recruitment and/or relative sapling abundance that can be potentially addressed through management.

In the language of the RAD framework, our work suggests opportunities to resist, accept, or direct ongoing changes in tree regeneration. Resistance strategies include actions that promote restoration of historical conditions as well as actions that encourage maintenance of current conditions (Schuurman et al., 2022). Our work highlights the value of improving oak regeneration as a resistance strategy that offers synergy between restoration, climate adaptation, and forest resilience goals. The increase of shade‐tolerant saplings across this region has been recognized as a key barrier to oak recruitment, with management strategies targeted at reducing their abundance viewed as a key component for increasing oak recruitment (Dey, 2014).

Resistance strategies to maintain current composition may be worthwhile in the case of species that are overabundant compared to the precolonial period and in active decline in the regeneration layer yet are resilient to likely future conditions. These species include sugar maple, red maple, black cherry (*Pr. serotina*), and aspen (*Populus* species). Red maple is an interesting case because it has increased throughout much of the eastern United States, particularly the Midwest, and its increase has been viewed as undesirable, particularly in oak‐hickory forests (Fei & Steiner, 2007). However, red maple may be a valuable component of northern temperate and southern boreal forests where we observed it to be in decline, as this species is resilient to climate change, pests, and disturbance. Low sapling recruitment of both sugar maple and oaks also suggests long‐term decreases in forest carbon stocks (Harris et al., 2024a), meaning that reversing regeneration declines for these species may have benefits for climate mitigation as well as climate adaptation goals.

Encouraging regeneration trends most consistent with an “accept” strategy were concentrated in northern and high‐elevation portions of the northeastern United States that support mixedwood or spruce fir forest. Increasing red spruce (*Pic. rubens*) regeneration, as observed here, suggests ongoing recovery of a “signature” species of the Acadian forest of northeastern North America often prioritized for its ecological and commercial value following over a century of selective harvesting and high‐intensity disturbances that favored balsam fir and shade‐intolerant hardwood species (Kelty & D'Amato, 2006; Noseworthy & Beckley, 2020). Increasing red spruce regeneration may also represent recovery from the effects of past soil acidification, which caused declines in red spruce over the late 20th century (Battles & Fahey, 2000), and which is still likely influencing the competitive balance between sugar maple and beech in northern hardwood forests (Zarfos et al., 2024). At the same time, declining balsam fir recruitment rates may portend decreases for a species that has long been encouraged by high‐intensity harvesting and natural disturbance yet is highly vulnerable to climate change, pests and disturbances (Collier et al., 2022; Noseworthy & Beckley, 2020).

Finally, the potential opportunity to direct regeneration trajectories toward novel compositions is exemplified by species like eastern hemlock (*Tsuga canadensis*), beech, and white ash (*Fraxinus americana*), which are already less abundant than they were historically and are heavily impacted by invasive forest pests and pathogens. Continued preservation and restoration efforts are vital for these tree species (D'Amato et al., 2023). Particularly in the short term, though, encouraging regeneration of species with overlapping ecosystem function and in some cases improved climate adaptation potential could help to maintain ecological integrity and counterbalance impacts of species loss to novel stressors. Nevertheless, such strategies may be limited where suitable replacement species do not exist in a local ecosystem or within the future climate niche of a given locale.

## CONCLUSIONS

Warnings that natural tree regeneration may be undesirable in terms of both abundance and composition in temperate and southern boreal forests (Cerioni et al., 2024; Miller & McGill, 2019) provide impetus for studies like ours that link regeneration patterns and trends to potential future outcomes, such as forest vulnerability to climate change, forest resilience, and compositional novelty. In our assessment of regeneration from 2003 to 2023 in the northeastern United States, we found trends consistent with forest maturation and recovery from intensive land use, but also trends consistent with novel drivers such as historically high deer populations and invasive species. At a regional scale, the regeneration patterns and trends that we observed suggest continued high vulnerability to climate change; decreased resilience to pests, pathogens and disturbance; and a shift toward novel species composition. However, the composition of small seedlings was more favorable for future climate and disturbance conditions than sapling composition and suggests opportunities to alter regeneration trajectories by addressing factors that limit seedling survival, including herbivory, light availability, and competition with understory vegetation. Considering how regeneration trajectories align with desired long‐term ecological, social, and cultural values for a given area can help prioritize where such interventions would be most useful (cf. Ontl et al., 2018).

## CONFLICT OF INTEREST STATEMENT

The authors declare no conflicts of interest.

## Supporting information


Appendix S1.

Appendix S2.

## Data Availability

Nationwide Forest Inventory (NFI) data were accessed from the Forest Inventory and Analysis Datamart by downloading the “Entire FIADB SQLite Database” from the “NFI Data” tab (https://apps.fs.usda.gov/fia/datamart/datamart.html). Output from the DISTRIB‐II model of the Climate Change Tree Atlas (Peters et al., 2019a) was used to assess current and project future climate suitability by tree species. Species trait scores used to characterize resilience were compiled from Matthews et al. (2011); these scores are publicly available from the USDA Forest Service Forest Ecosystem Atlas Tree Atlas and Bird Atlas Data Archive at https://experience.arcgis.com/experience/fbfad910532e4ae18fa3fe5af6e9260f. Historical tree composition data from early colonial land surveys (Thompson & Cogbill, 2023) are available in the Environmental Data Initiative at https://doi.org/10.6073/pasta/4d164206805433114d21b8a51dc0e91e. Code to reproduce the analysis and a table of the compiled species trait scores are provided in Harris et al. (2026) in Figshare at https://doi.org/10.6084/m9.figshare.29341526.
