# Supplementary material for: Tree regeneration trends in forests of the northeastern USA and their implications for resilience and restoration
Source: Ecol Appl. 2026 Jul 28;36(5):e70288. doi: 10.1002/eap.70288 (PMC13413246; doi:10.1002/eap.70288)
Supplement: Supplementary file 1 — Appendix S1. [file EAP-36-e70288-s002.pdf]

Appendix S1

Tree regeneration trends in forests of the northeastern USA and their implications for resilience and restoration

Lucas B. Harris, Melissa A. Pastore, Anthony W. D'Amato

Ecological Applications

Appendix S1: Table S1. Common tree species for which trends in abundance were analyzed with the number and percentage of forest inventory subplot measurements from 2003 to 2023 in which a live individual of the species was present. Species palatability to white-tailed deer (*Odocoileus virginiana*) is also shown, as compiled from the literature by Harris et al. (2025).

Species	Subplots	Percentage	Patalability
Acer rubrum	65551	55.2	high
Abies balsamea	45210	38.1	low
Acer saccharum	38180	32.2	high
Fagus grandifolia	35873	30.2	low
Betula alleghaniensis	34127	28.7	high
Picea rubens	33957	28.6	low
Fraxinus americana	24274	20.4	high
Pinus strobus	23863	20.1	moderate
Acer pensylvanicum	23670	19.9	low
Betula papyrifera	23567	19.8	high
Tsuga canadensis	22983	19.4	moderate
Quercus rubra	16305	13.7	high
Prunus serotina	13452	11.3	low
Thuja occidentalis	12900	10.9	high
Ostrya virginiana	9453	8	low
Populus tremuloides	9396	7.9	low
Acer spicatum	7627	6.4	
Betula lenta	7089	6	high
Amelanchier spp.	7027	5.9	
Picea glauca	6640	5.6	low
Quercus alba	4391	3.7	high
Prunus pensylvanica	4383	3.7	high
Betula populifolia	3975	3.3	moderate
Populus grandidentata	3823	3.2	low
Picea mariana	3818	3.2	low
Ulmus americana	3754	3.2	
Fraxinus nigra	3062	2.6	high
Sorbus americana	2747	2.3	

Appendix S1: Table S2. Species-level trends in absolute and relative abundance of trees (≥12.7 cm DBH) using basal area (as in Table 2) and stem density, indicated by Sen's slope with corrected p-values. Significant trends ($p < 0.05$) are in bold.

	Tree basal area (m^2 ha^{-1} $year^{-1}$)		Tree density (stems ha^{-1} $year^{-1}$)	
Species	Absolute	Relative (%)	Absolute	Relative (%)
Abies balsamea	**0.04 (<0.001)**	**0.15 (0.003)**	**1.8 (<0.001)**	**0.18 (0.004)**
Acer pensylvanicum	**0.00076 (0.004)**	0.0035 (0.513)	**0.043 (0.002)**	**0.0085 (0.006)**
Acer rubrum	0.016 (0.609)	-0.043 (0.857)	-0.0068 (1)	-0.044 (0.8)
Acer saccharum	-0.0015 (1)	-0.067 (0.095)	-0.18 (1)	**-0.073 (0.048)**
Acer spicatum	0 (1)	0 (1)	0 (1)	0 (1)
Betula alleghaniensis	**0.022 (<0.001)**	**0.082 (<0.001)**	**0.47 (<0.001)**	**0.083 (<0.001)**
Betula papyrifera	-0.0024 (1)	**-0.042 (0.01)**	-0.049 (1)	**-0.035 (0.027)**
Betula populifolia	0.00025 (1)	0.0013 (1)	0.021 (0.423)	0.0021 (1)
Fagus grandifolia	**0.0096 (0.043)**	0.04 (0.307)	**0.4 (<0.001)**	**0.075 (0.01)**
Picea rubens	**0.031 (0.003)**	0.088 (0.274)	**0.84 (<0.001)**	0.076 (0.376)
Pinus strobus	0.0077 (1)	-0.024 (1)	-0.21 (0.091)	-0.046 (0.056)
Populus tremuloides	-0.00026 (1)	**-0.02 (0.012)**	-0.016 (1)	**-0.022 (0.002)**
Prunus pensylvanica	-0.00016 (1)	-0.004 (0.095)	-0.0073 (1)	-0.0051 (0.108)
Prunus serotina	-0.0017 (1)	-0.024 (0.205)	-0.078 (0.195)	-0.024 (0.078)
Quercus alba	-0.0023 (0.132)	**-0.017 (0.005)**	**-0.063 (0.035)**	**-0.019 (0.023)**
Quercus rubra	**0.012 (0.037)**	0.0019 (1)	-0.056 (1)	-0.0081 (1)
Thuja occidentalis	0.011 (1)	0.0058 (1)	0.075 (1)	-0.0057 (1)
Ulmus americana	-0.00076 (1)	-0.015 (0.126)	-0.037 (0.632)	-0.016 (0.122)

Appendix S1: Table S3. Mean abundance of seedlings 5–15 cm and 15−30 cm tall (stems ha^{-1}) at the first (2012–2018) and second (2018–2023) measurement cycle for Regeneration Indicator plots, and corrected p-values from Wilcoxon signed-rank tests.

	5−15 cm tall			15−30 cm tall		
Species	Time 1	Time 2	p-value	Time 1	Time 2	p-value
Abies balsamea	1026.056	983.6655	1	565.7068	483.1563	1
Acer pensylvanicum	441.7571	673.5432	0.01	173.2818	142.7902	1
Acer rubrum	2986.446	3064.535	0.41	644.291	732.7912	1
Acer saccharum	1532.267	2196.39	0.012	511.4168	483.4042	1
Acer spicatum	165.8448	147.7481	0.58	133.6179	104.6136	1
Betula alleghaniensis	156.6725	145.0213	0.11	119.2397	119.2397	1
Betula papyrifera	57.7606	35.20174	1	42.88663	43.38243	1
Betula populifolia	13.63448	24.29416	1	6.197489	28.26055	1
Fagus grandifolia	279.3828	277.8954	1	200.055	217.9037	1
Picea rubens	226.3323	285.5803	1	190.139	200.055	1
Pinus strobus	238.4794	240.2147	1	153.6977	77.09677	1
Populus tremuloides	25.28576	22.80676	1	41.64713	34.21014	1
Prunus pensylvanica	12.89078	7.684887	1	28.01265	25.78156	1
Prunus serotina	92.96234	132.6263	1	57.5127	49.33202	0.41
Quercus alba	8.428586	2.231096	0.16	17.60087	15.61767	1
Quercus rubra	121.7187	115.7691	1	98.91193	93.70604	1
Thuja occidentalis	162.87	124.6935	1	135.6011	116.017	1
Ulmus americana	3.470594	7.932787	1	9.420184	10.41178	1

Appendix S1: Table S4. Mean (standard deviation) of relative abundance (%) from 2018 to 2023 by species by tree, sapling, sapling recruitment and small seedling (5–15 cm and 15−30 cm tall) density for the 28 common tree species analyzed for temporal trends.

Species	Tree	Sapling	Sapling recruitment	5-15 cm tall	15-30 cm tall
Abies balsamea	9.7 (21.8)	17.5 (32.8)	17.6 (35.8)	10.3 (24)	12.5 (26.8)
Acer pensylvanicum	0.5 (3.9)	4.9 (18.2)	6.4 (22.8)	6.3 (17.4)	4.5 (16.1)
Acer rubrum	15.6 (24.2)	10.5 (26.4)	8.6 (25.9)	31.4 (36.1)	14.4 (27.3)
Acer saccharum	10.7 (23)	6.8 (22.6)	3.3 (16.9)	15.5 (29.9)	8.6 (22.5)
Acer spicatum	0 (0.4)	0.4 (5.4)	0.5 (6.8)	1.9 (10.4)	2.9 (13.5)
Amelanchier spp.	0.1 (2)	0.6 (6.8)	0.5 (6.9)	1.1 (7.2)	1.9 (9.8)
Betula alleghaniensis	6.5 (15.6)	4.5 (17.5)	4.3 (18.4)	1.5 (8.1)	2.6 (11.6)
Betula lenta	1.6 (8.1)	1.4 (10.7)	1.4 (11.2)	0.2 (3.1)	0.4 (5.2)
Betula papyrifera	3 (9.9)	2 (10.3)	1.4 (10.1)	0.6 (5.2)	0.8 (5.7)
Betula populifolia	0.4 (3.9)	0.8 (7.3)	1.1 (9.4)	0.1 (3.3)	0.2 (3.8)
Fagus grandifolia	8.2 (19.6)	16.7 (34.3)	18.1 (37.2)	4.7 (17.4)	10.4 (27.4)
Fraxinus americana	3.3 (12)	2.9 (14.9)	3.6 (17.7)	6.7 (20.4)	8.6 (23)
Fraxinus nigra	0.4 (4)	0.7 (7)	0.8 (8.7)	0 (0.7)	0.3 (4.2)
Ostrya virginiana	0.8 (5.5)	2.5 (14)	1.9 (13.1)	0.9 (6.4)	1.6 (9.7)
Picea glauca	0.8 (6.3)	0.6 (6.6)	0.7 (8.1)	0.3 (4.6)	0.4 (4.9)
Picea mariana	1.1 (8.9)	1.2 (9.8)	1.1 (9.9)	0.5 (6.6)	0.9 (8.4)
Picea rubens	6.9 (17.7)	8.6 (23.5)	10.8 (29.2)	2.2 (10.3)	3.9 (14.9)
Pinus strobus	5.5 (16.6)	2.1 (12.6)	2.8 (15.3)	2.9 (12.5)	2.6 (12.5)
Populus grandidentata	0.7 (5.7)	0.3 (4.4)	0.4 (5.7)	0.1 (2.2)	0.2 (4.1)
Populus tremuloides	1.4 (8.2)	0.8 (7.5)	1.4 (11)	0.6 (6.6)	1.6 (10.8)
Prunus pensylvanica	0.2 (2.4)	0.4 (5)	0.5 (6)	0.2 (3.4)	0.4 (4.5)
Prunus serotina	1.7 (8.5)	0.9 (8.1)	0.9 (8.6)	1.9 (10.3)	1.8 (10.4)
Quercus alba	0.7 (5.1)	0.2 (3.9)	0.1 (2.5)	0.1 (2.7)	0.6 (6.3)
Quercus rubra	3.5 (12.1)	1 (8.4)	1 (9)	2.4 (11.9)	3.2 (14.1)
Sorbus americana	0 (0.7)	0.1 (2.4)	0.1 (2.9)	0.4 (4.4)	0.8 (7.2)
Thuja occidentalis	2.8 (12.8)	1.8 (11.3)	2.5 (14.7)	1.9 (10.3)	3.4 (15.2)
Tsuga canadensis	6.4 (17.3)	4 (17.7)	3.2 (16.9)	1.2 (7.2)	2.6 (13.4)
Ulmus americana	0.5 (4.7)	0.6 (6.9)	0.4 (6.1)	0.3 (4.8)	0.5 (6)

Appendix S1: Table S5. Linear trend estimates (Sen's slope estimator) for annual percentage within different categories of projected change in habitat suitability based on relative abundance among trees, saplings and sapling recruits. For example, the "large decrease" category refers to species and locations forecast to experience >50% declines in habitat suitability due to climate change by the end of the century. Holm-corrected p-values are shown with significant <0.05 values in bold.

Category	Change category	Trend (% yr^{-1})	p-value
Tree	large decrease	-0.03	1
Tree	decrease	-0.04	0.79
Tree	no change	-0.01	1
Tree	increase	0.04	0.82
Tree	large increase	0.05	0.28
Sapling	large decrease	-0.06	0.62
Sapling	decrease	-0.04	0.6
Sapling	no change	0.00	1
**Sapling**	**increase**	**0.07**	**0.017**
Sapling	large increase	0.08	0.1
**Sapling recruitment**	**large decrease**	**-0.58**	**0.011**
Sapling recruitment	decrease	-0.05	1
Sapling recruitment	no change	0.24	0.055
**Sapling recruitment**	**increase**	**0.18**	**0.018**
Sapling recruitment	large increase	0.22	0.055

Appendix S1: Table S6. Estimated annual trends in relative abundance (% of stems within a subplot, averaged annually over the northeastern USA) by genus/group of species (with corrected p-values, significant trends in bold) from plots within the 423 towns included in the witness tree dataset used to assess pre-colonial forest composition.

Name	Tree	Sapling	Sapling recruitment
Ash	-0.028 (1)	-0.018 (1)	-0.015 (1)
Basswood	-0.005 (1)	-0.007 (1)	0 (1)
Beech	**0.131 (0.002)**	**0.370 (<0.001)**	0.489 (0.133)
Birch	0.054 (0.67)	-0.018 (1)	0.079 (1)
Black gum	0 (1)	0.003 (1)	0 (1)
Cedar	-0.01 (1)	0.007 (1)	-0.002 (1)
Cherry	-0.026 (0.358)	**-0.064 (0.004)**	-0.017 (1)
Chestnut	0 (1)	-0.001 (1)	0 (0.418)
Elm	-0.013 (0.522)	-0.024 (0.273)	0.016 (1)
Fir	0.083 (0.226)	0.078 (1)	-0.318 (1)
Hemlock	0.001 (1)	-0.061 (0.09)	-0.06 (1)
Hickory	-0.003 (1)	-0.01 (1)	-0.005 (1)
Hornbeam	-0.01 (1)	0.05 (1)	0.056 (1)
Magnolia	0 (1)	0 (1)	0 (1)
Maple	-0.064 (1)	**-0.235 (0.005)**	-0.385 (0.548)
Oak	-0.064 (0.138)	-0.021 (1)	0.013 (1)
Pine	-0.073 (0.401)	-0.082 (0.065)	-0.073 (1)
Poplar	-0.014 (1)	-0.037 (0.207)	0.034 (1)
Spruce	0.062 (1)	**0.139 (0.005)**	0.307 (0.952)
Tamarack	-0.003 (1)	0.001 (1)	0 (1)
Tulip	0 (1)	0 (1)	0 (1)
Other	-0.026 (0.67)	-0.038 (1)	-0.152 (0.813)

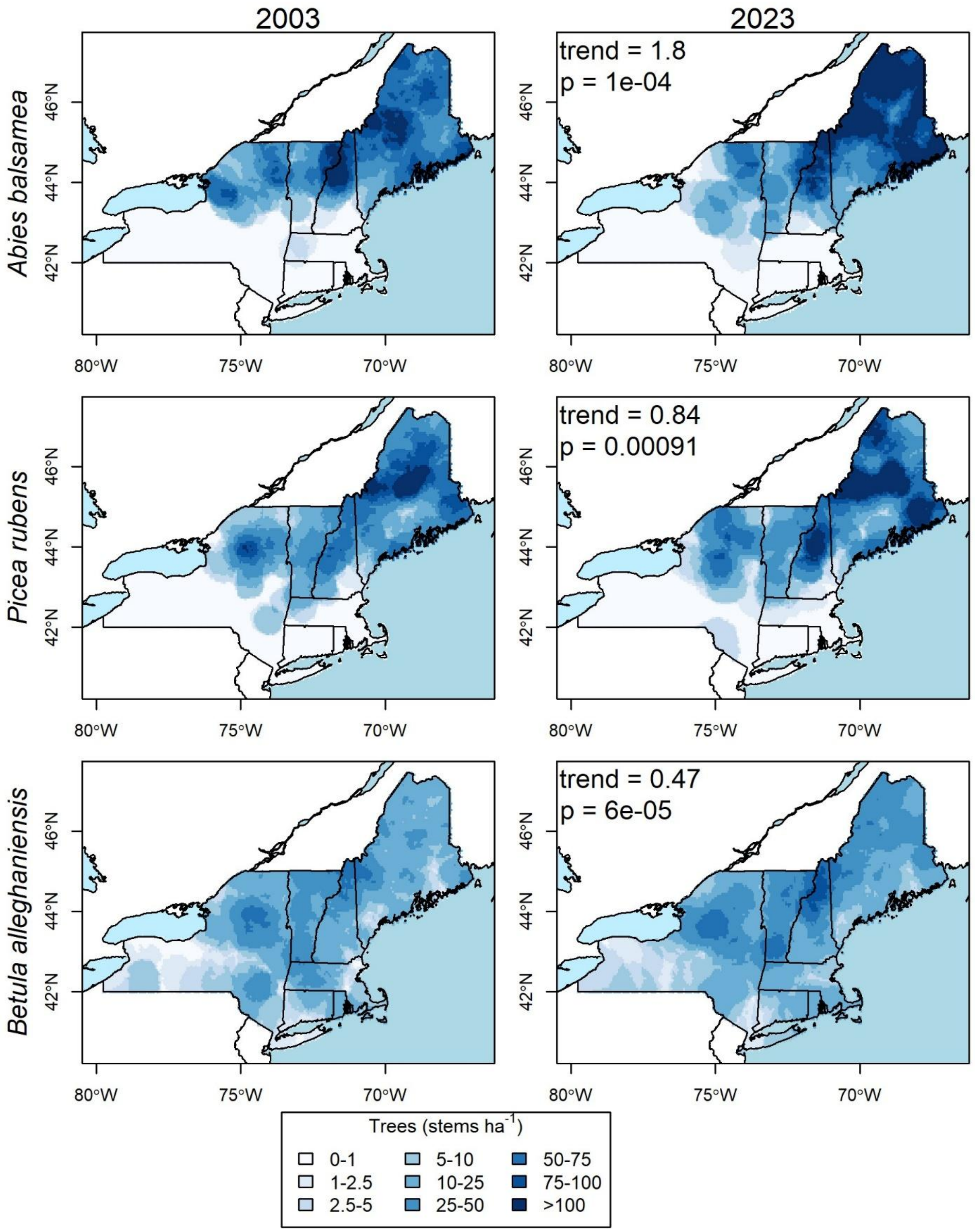

Appendix S1: Figure S1. Interpolated maps of live tree density at the start and end years of the analysis (2003 and 2023) for *Abies balsamea* (balsam fir), *Picea rubens* (red spruce) and *Betula alleghaniensis* (yellow birch). Estimated annual trends and p-values from Sen's slope tests are shown at the upper left of the year 2023 panels.

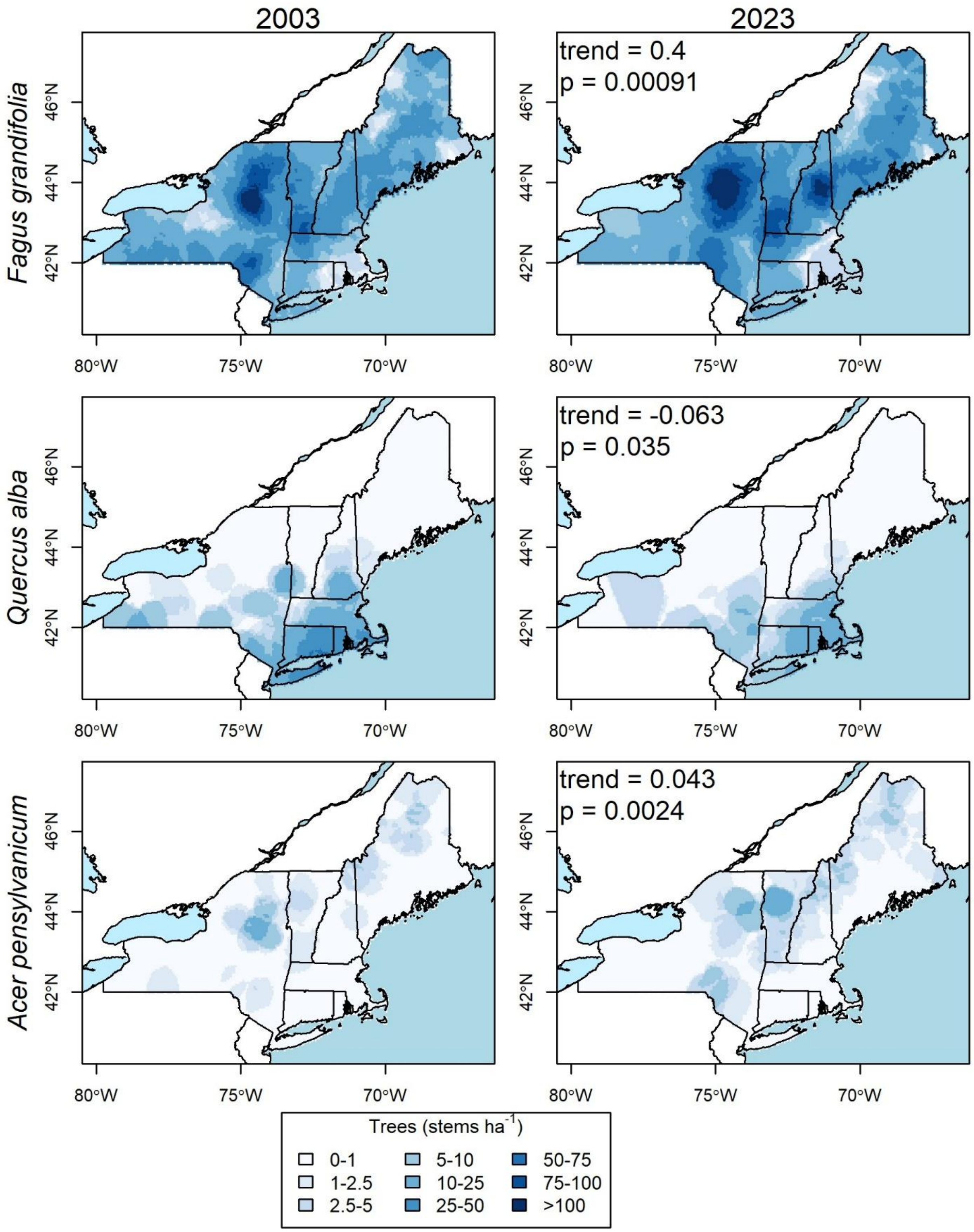

Appendix S1: Figure S2. Interpolated maps of live tree density at the start and end years of the analysis (2003 and 2023) for *Fagus grandifolia* (American beech), *Quercus alba* (white oak), and *Acer pensylvanicum* (striped maple). Estimated annual trend and p-value from Sen's slope tests are shown at the upper left of the year 2023 panels.

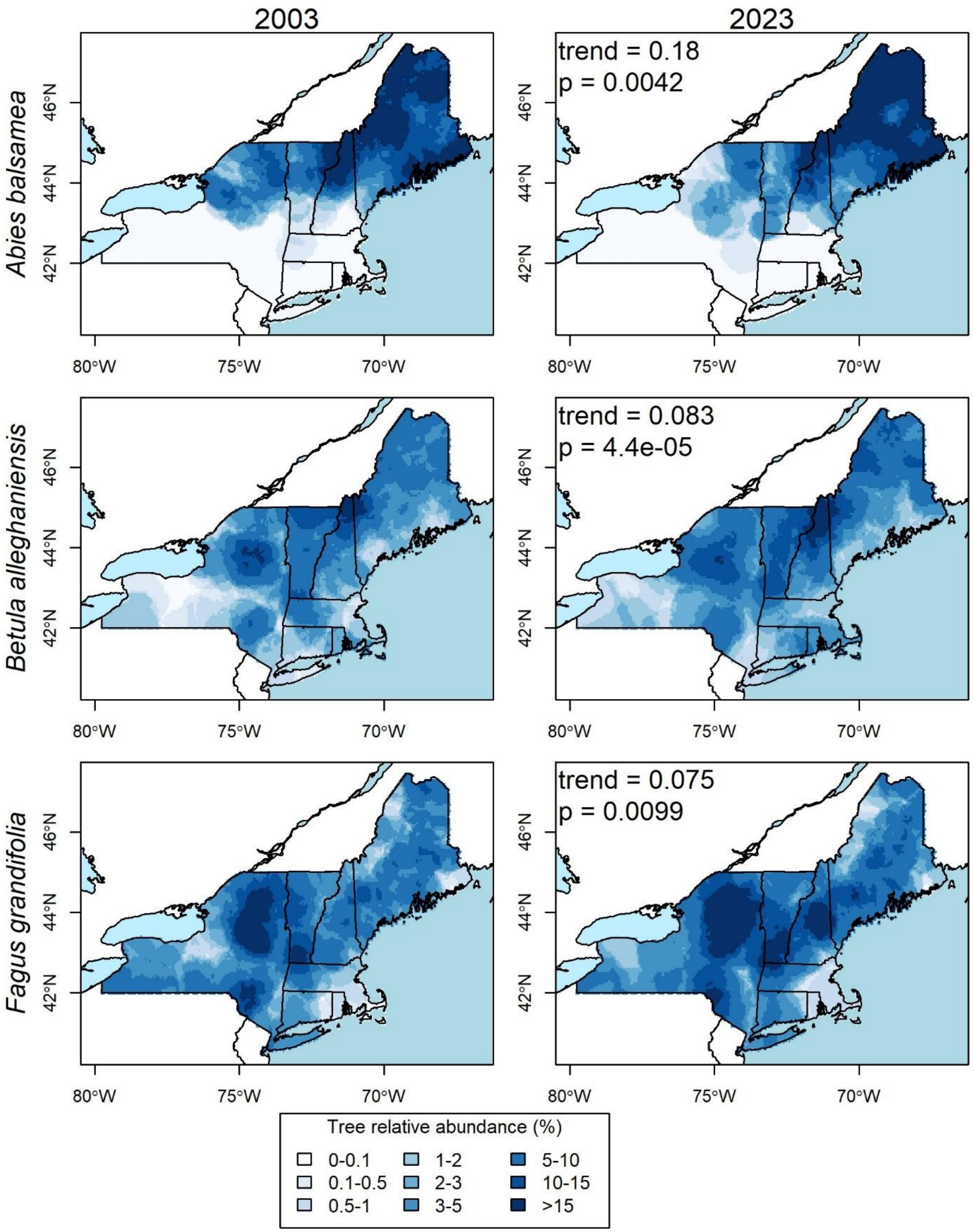

Appendix S1: Figure S3. Interpolated maps of relative tree abundance by density at the start and end years of the analysis (2003 and 2023) for *Abies balsamea* (balsam fir), *Betula alleghaniensis* (yellow birch) and *Fagus grandifolia* (American beech). Estimated annual trends and p-values from Sen's slope tests are shown at the upper left of the year 2023 panels.

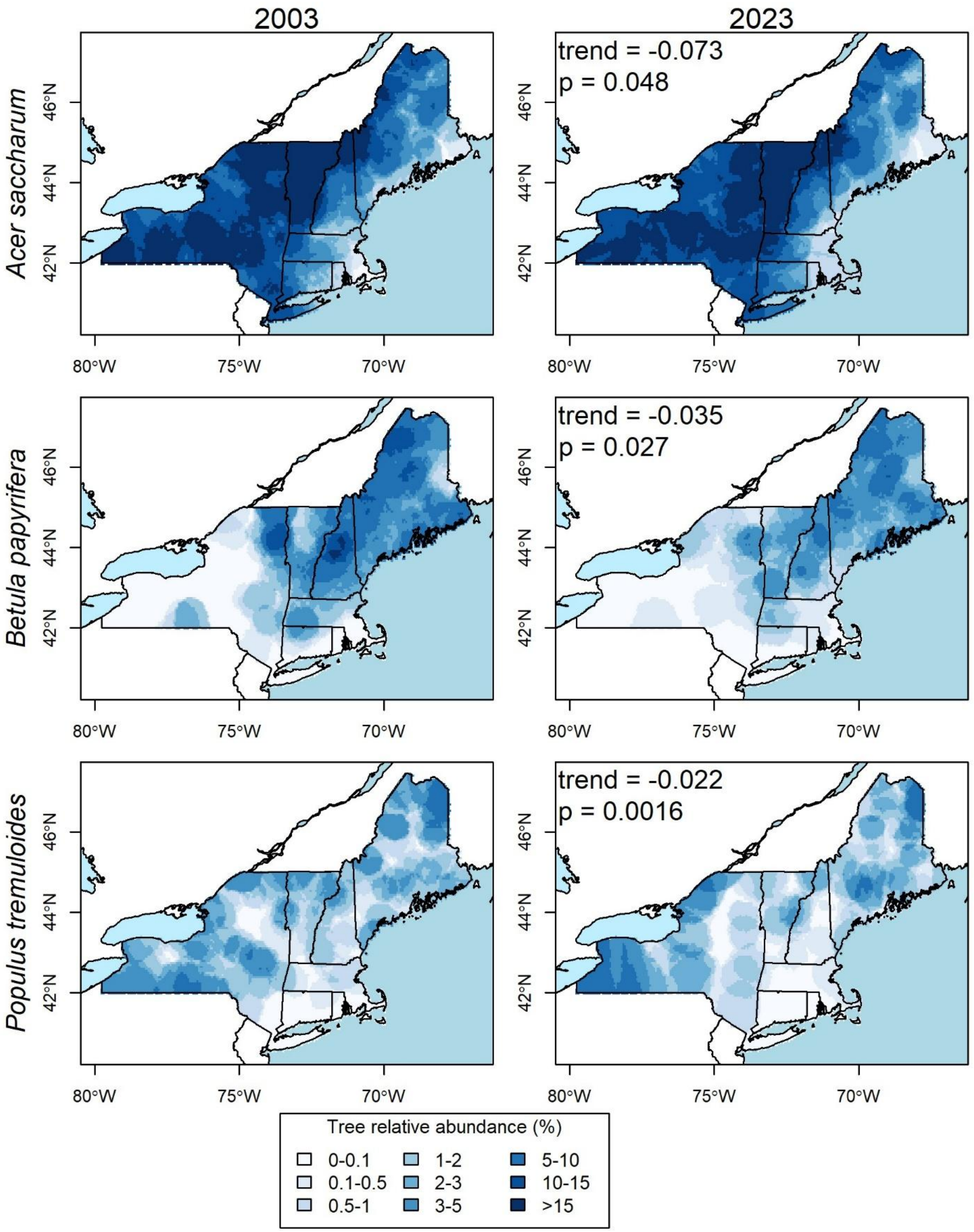

Appendix S1: Figure S4. Interpolated maps of relative tree abundance by density at the start and end years of the analysis (2003 and 2023) for *Acer saccharum* (sugar maple), *Betula papyrifera* (paper birch) and *Populus tremuloides* (quaking aspen). Estimated annual trends and p-values from Sen's slope tests are shown at the upper left of the year 2023 panels.

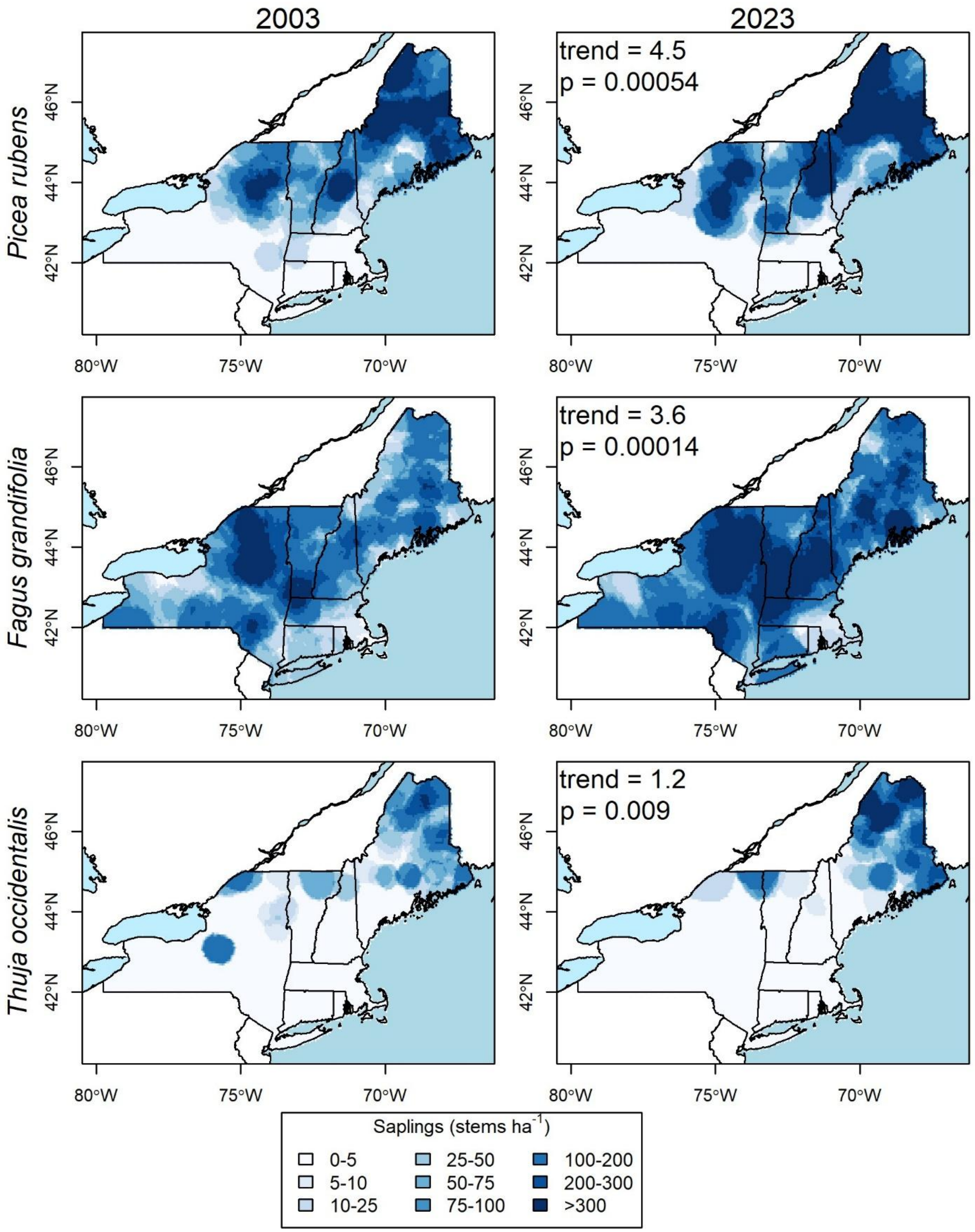

Appendix S1: Figure S5. Interpolated maps of absolute sapling abundance at the start and end years of the analysis (2003 and 2023) for *Picea rubens* (red spruce), *Fagus grandifolia* (beech) and *Thuja occidentalis* (northern white-cedar). Estimated annual trends and p-values from Sen's slope tests are shown at the upper left of the year 2023 panels.

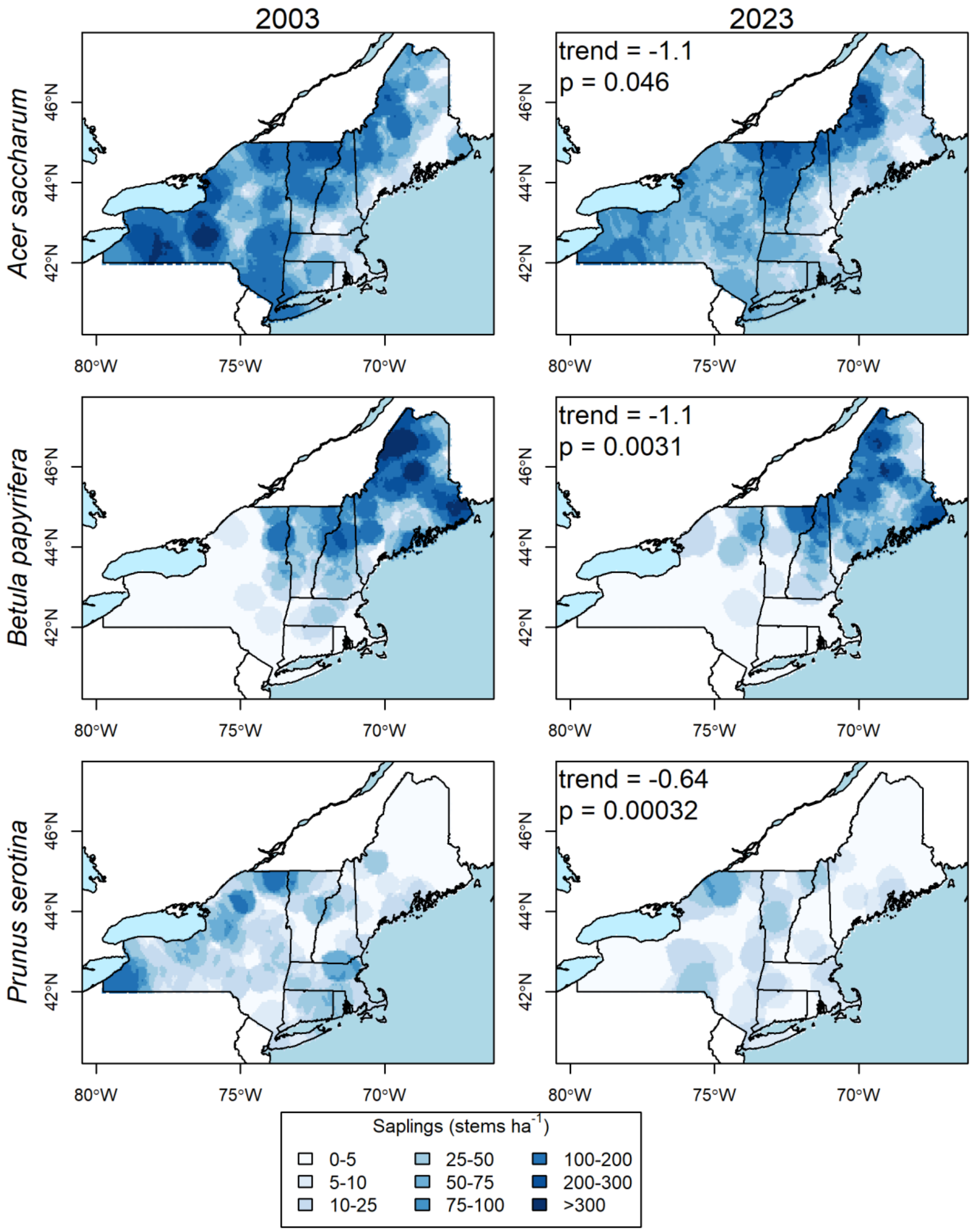

Appendix S1: Figure S6. Interpolated maps of absolute sapling abundance at the start and end years of the analysis (2003 and 2023) for *Acer saccharum* (sugar maple), *Betula papyrifera* (paper birch), *Prunus serotina* (black cherry) and *Prunus pensylvanica* (pin cherry). Estimated annual trends and p-values from Sen's slope tests are shown at the upper left of the year 2023 panels.

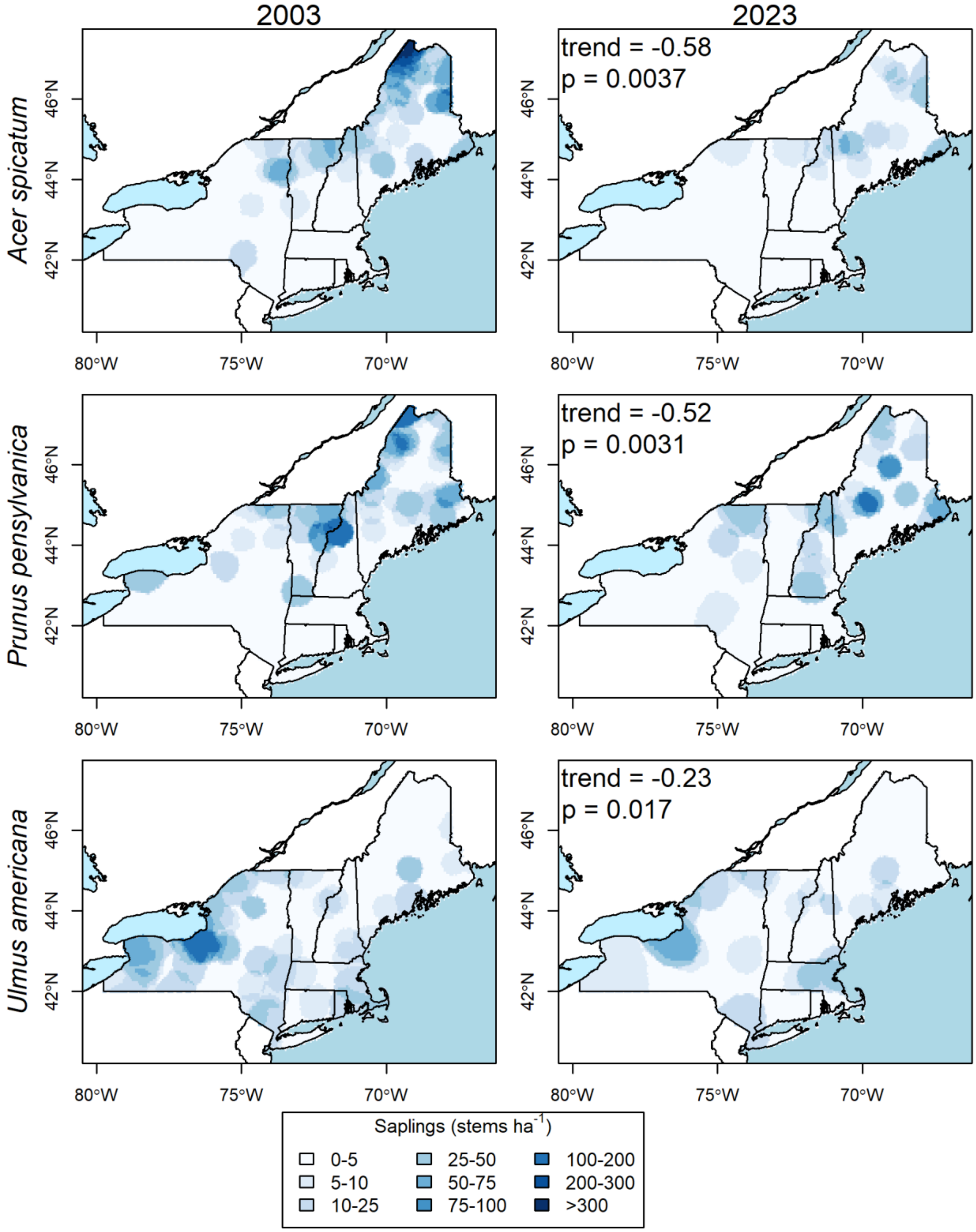

Appendix S1: Figure S7. Interpolated maps of absolute sapling abundance at the start and end years of the analysis (2003 and 2023) for *Acer spicatum* (mountain maple), *Prunus pensylvanica* (pin cherry) and *Ulmus americana* (American elm). Estimated annual trends and p-values from Sen's slope tests are shown at the upper left of the year 2023 panels.

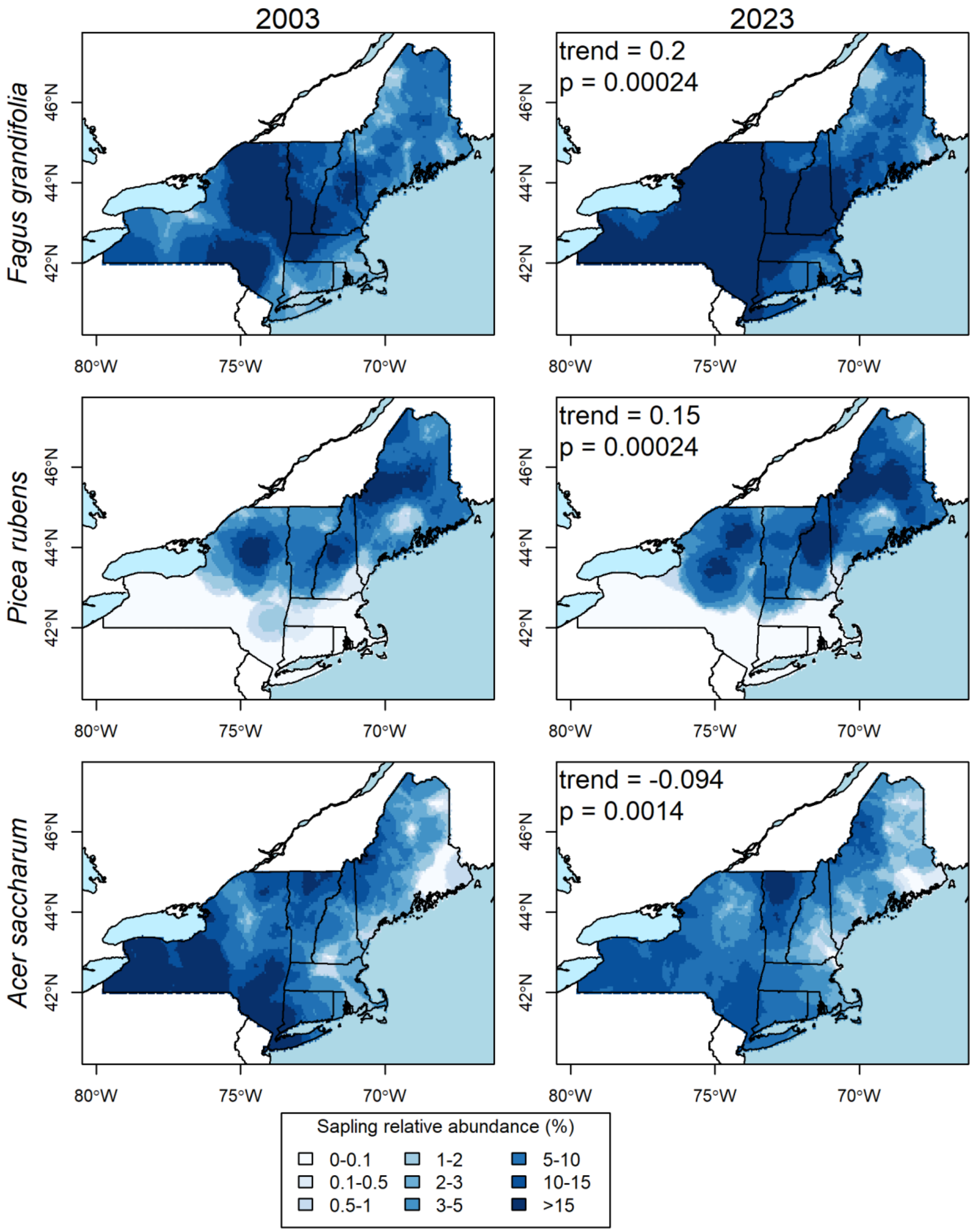

Appendix S1: Figure S8. Interpolated maps of relative sapling abundance at the start and end years of the analysis (2003 and 2023) for *Fagus grandifolia* (beech), *Picea rubens* (red spruce) and *Acer saccharum* (sugar maple). Estimated annual trends and p-values from Sen's slope tests are shown at the upper left of the year 2023 panels.

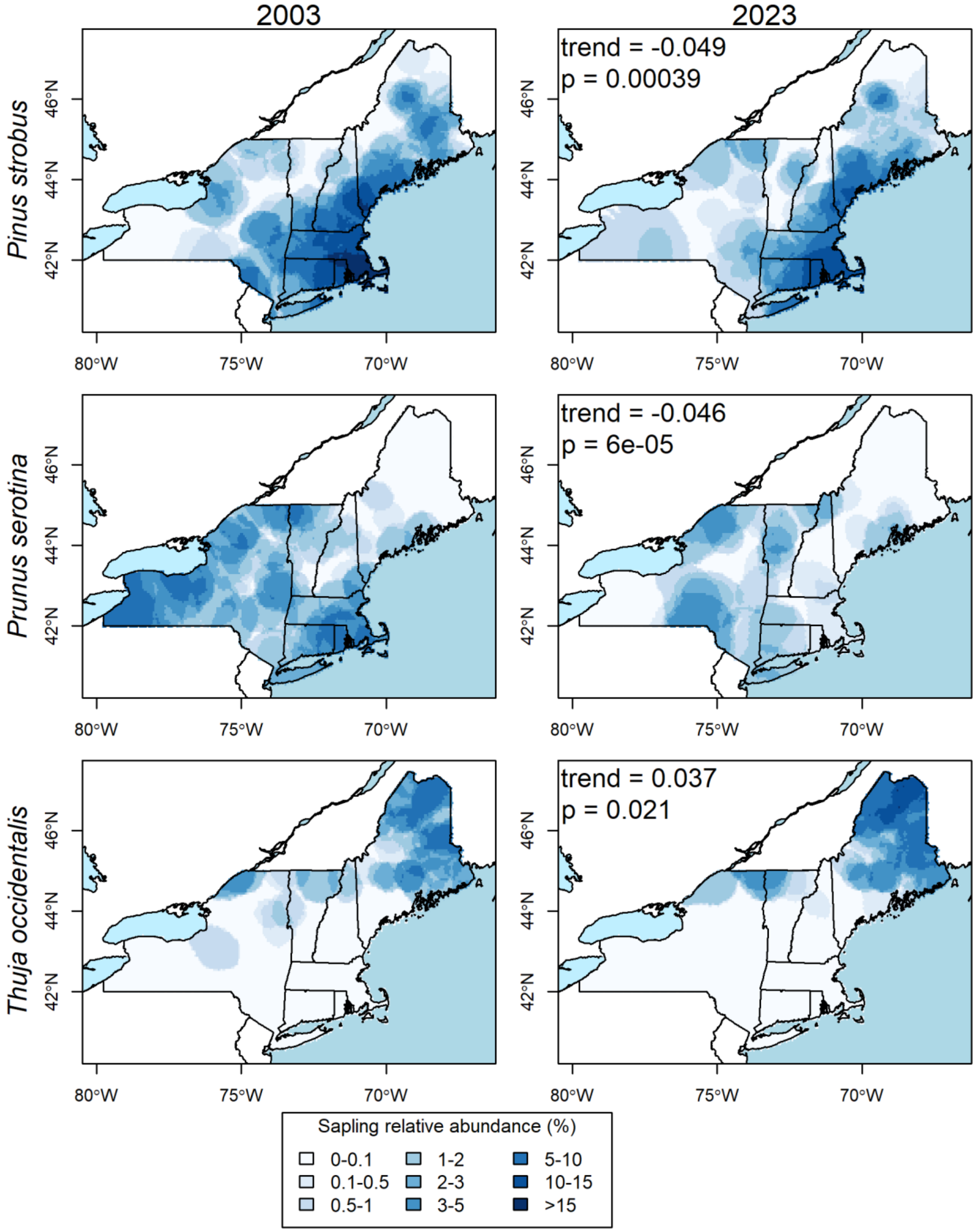

Appendix S1: Figure S9. Interpolated maps of relative sapling abundance at the start and end years of the analysis (2003 and 2023) for *Pinus strobus* (white pine), *Prunus serotina* (black cherry) and *Thuja occidentalis* (northern white cedar). Estimated annual trends and p-values from Sen's slope tests are shown at the upper left of the year 2023 panels.

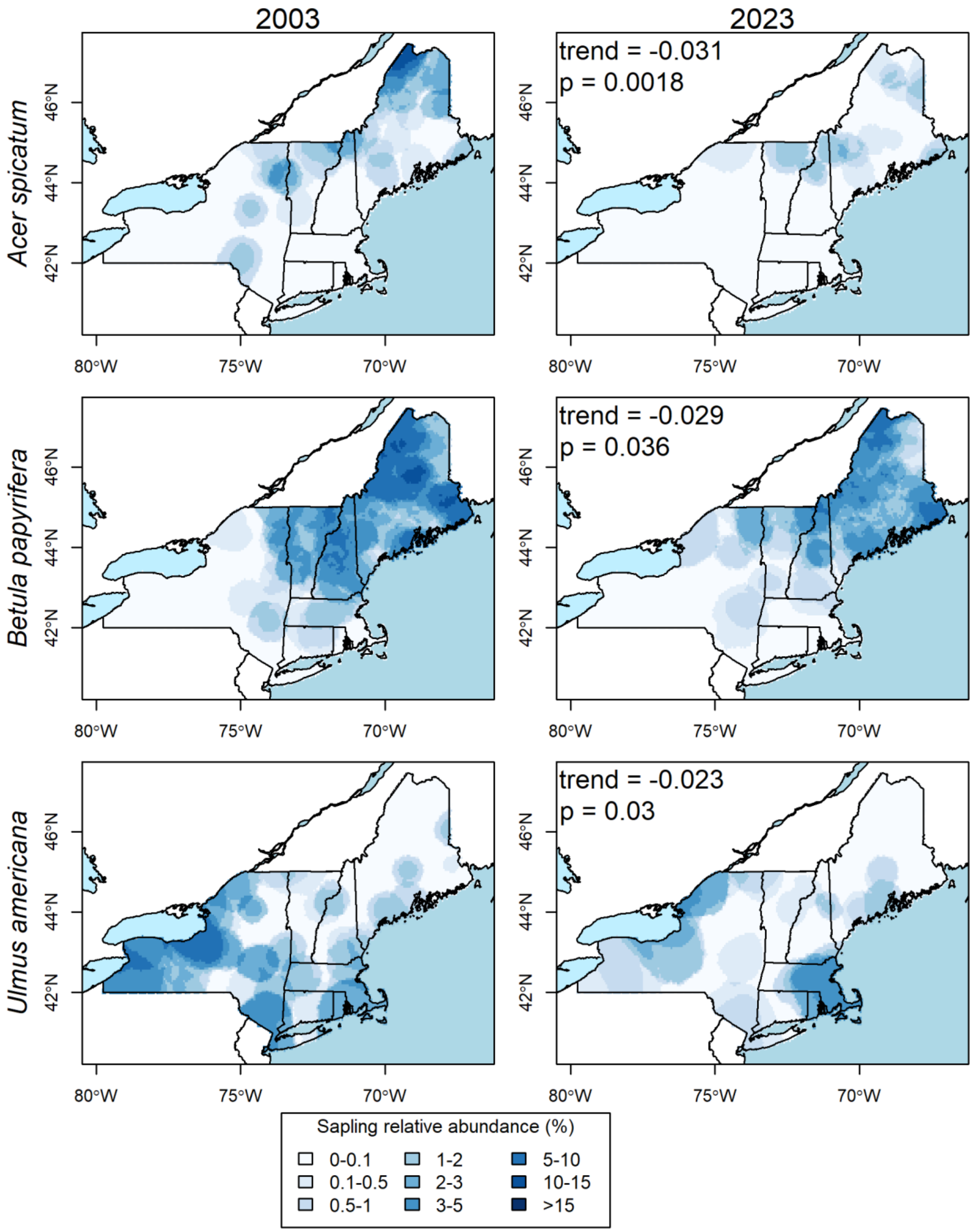

Appendix S1: Figure S10. Interpolated maps of relative sapling abundance at the start and end years of the analysis (2003 and 2023) for *Acer spicatum* (mountain maple), *Betula papyrifera* (paper birch) and *Ulmus americana* (American elm). Estimated annual trends and p-values from Sen's slope tests are shown at the upper left of the year 2023 panels.

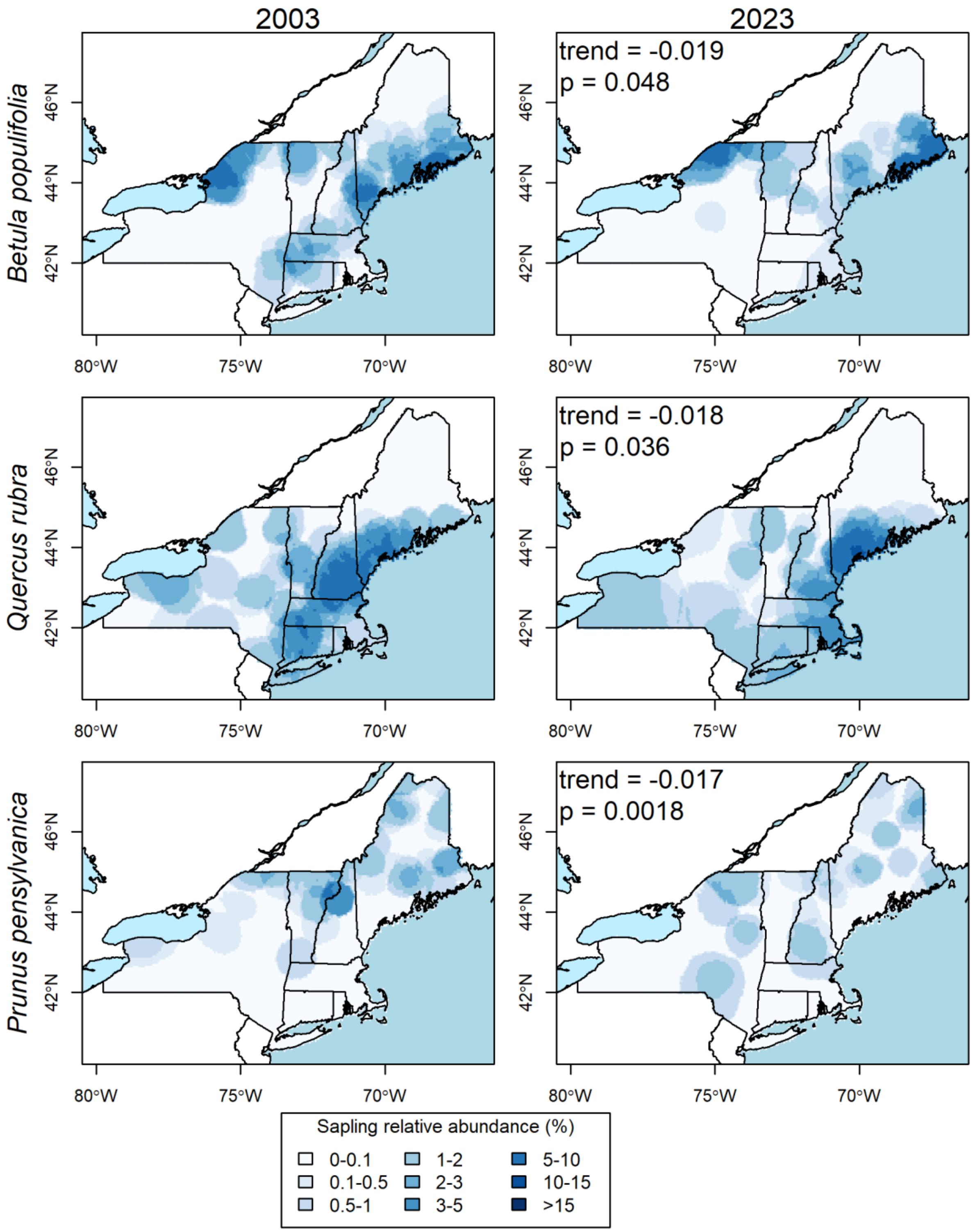

Appendix S1: Figure S11. Interpolated maps of relative sapling abundance at the start and end years of the analysis (2003 and 2023) for *Betula populifolia* (gray birch), *Quercus rubra* (northern red oak) and *Prunus pensylvanica* (pin cherry). Estimated annual trends and p-values from Sen's slope tests are shown at the upper left of the year 2023 panels.

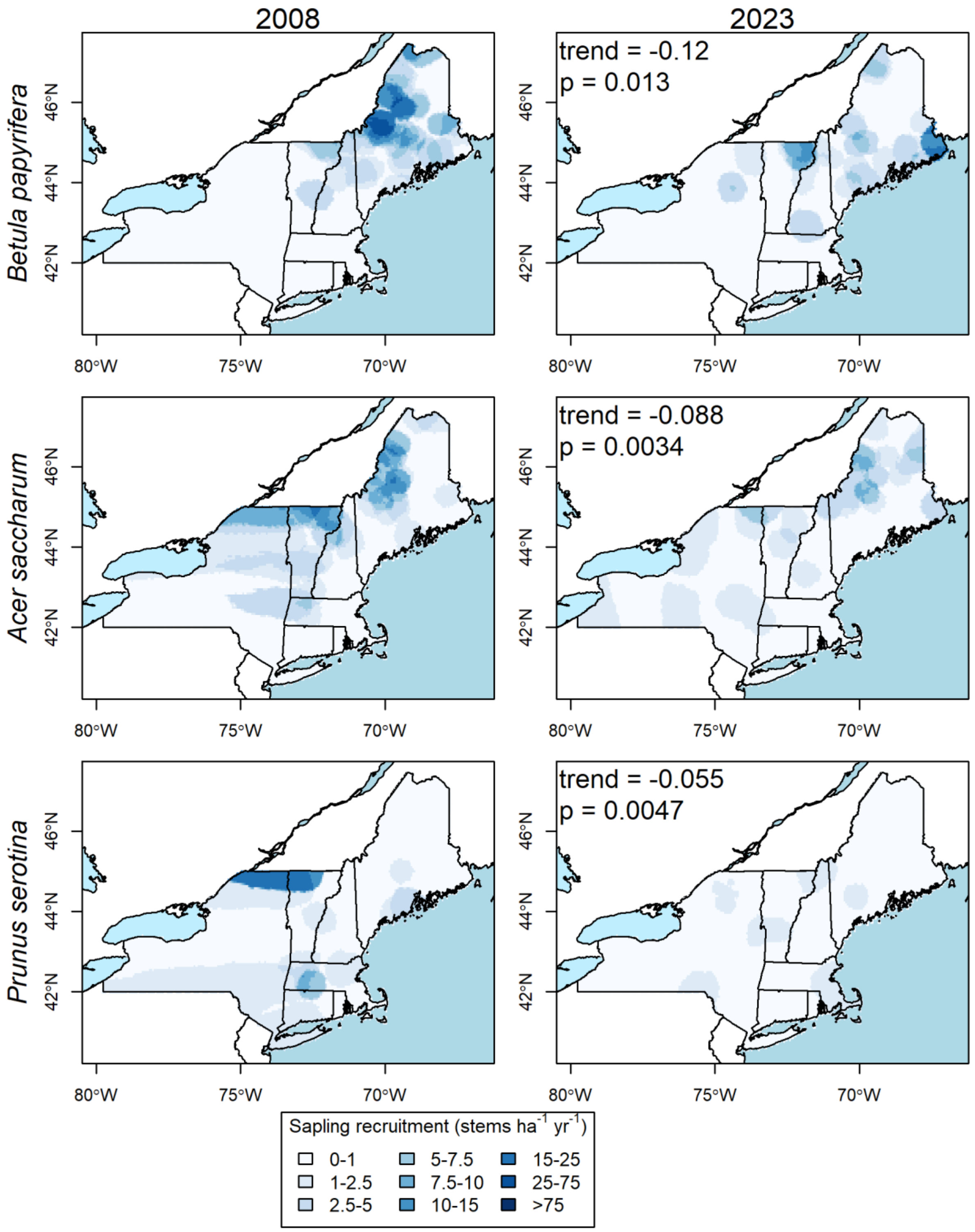

Appendix S1: Figure S12. Interpolated maps of sapling recruitment rates at the start and end years of the analysis (2008 and 2023) for *Betula papyrifera* (paper birch), *Acer saccharum* (sugar maple) and *Prunus serotina* (black cherry). Estimated annual trends and p-values from Sen's slope tests are shown at the upper left of the year 2023 panels.

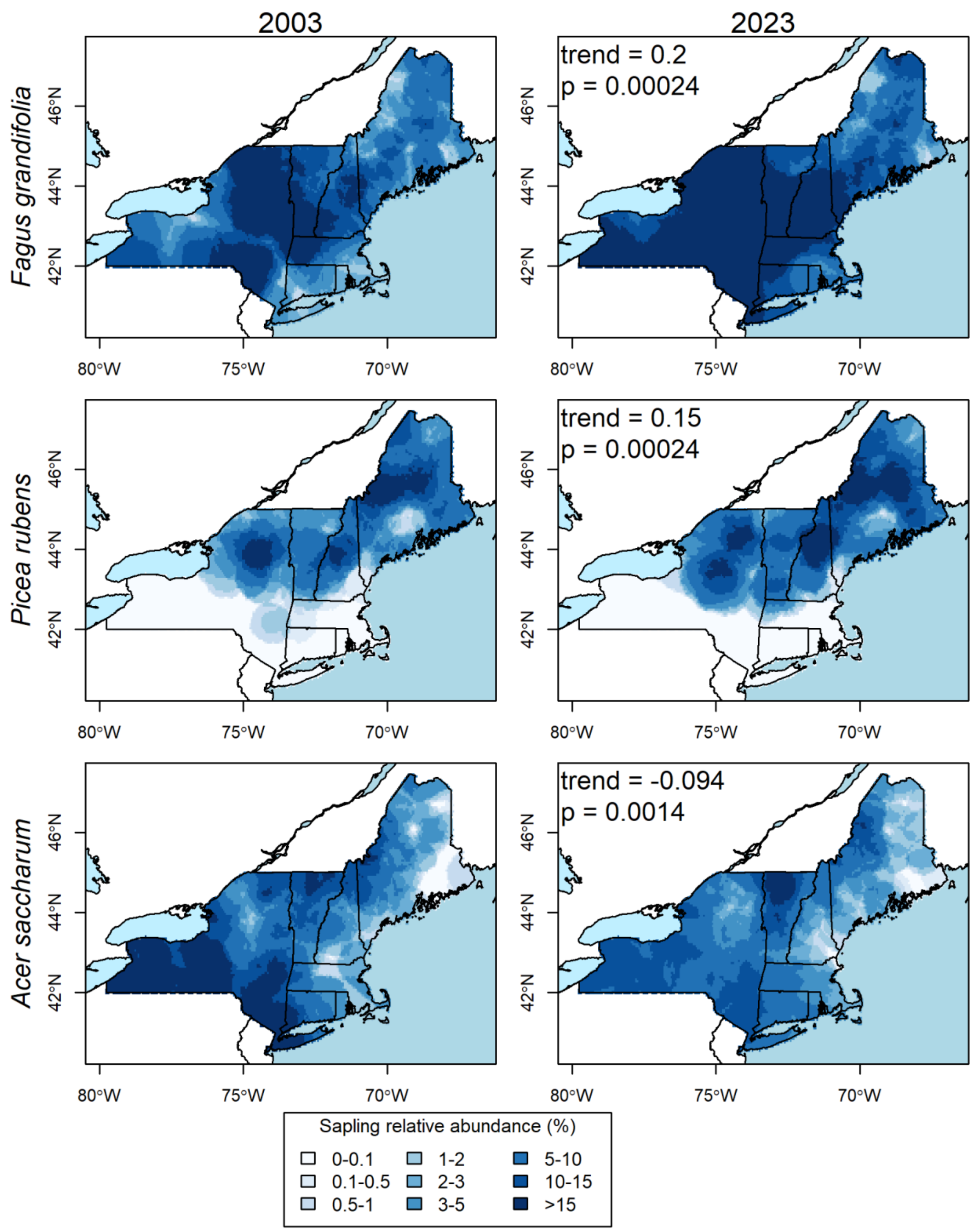

Appendix S1: Figure S13. Interpolated maps of relative abundance of recruited saplings at the start and end years of the analysis (2008 and 2023) for *Fagus grandifolia* (American beech), *Picea rubens* (red spruce) and *Acer saccharum* (sugar maple). Estimated annual trends and p-values from Sen's slope tests are shown at the upper left of the year 2023 panels.
