# Supplementary material for: Tree regeneration trends in forests of the northeastern USA and their implications for resilience and restoration
Source: Ecol Appl. 2026 Jul 28;36(5):e70288. doi: 10.1002/eap.70288 (PMC13413246; doi:10.1002/eap.70288)
Supplement: Supplementary file 2 — Appendix S2. [file EAP-36-e70288-s001.pdf]

Appendix S2

Tree regeneration trends in forests of the northeastern USA and their implications for resilience and restoration

Lucas B. Harris, Melissa A. Pastore, Anthony W. D'Amato

Ecological Applications

Section S1: Modeling sapling recruitment

We followed the methodology developed by Harris et al. (2022) and elaborated by Harris et al. (2024) to predict the presence of sapling recruitment in Time 2 based on abundance in Forest Inventory and Analysis (FIA) Regeneration Indicator (RI) seedling height classes in Time 1 as well as other covariates from the FIA database and ancillary geospatial datasets (Table S1).

We developed individual models for the 11 species with ≥15 subplots in which sapling recruitment was present at Time 2, as well as a model for all other species combined (Table S2). A Boosted Regression Tree (Elith et al., 2008) framework was used, implemented via the "dismo" R package (Hijmans et al., 2021) with 10-fold cross-validation, a learning rate of 0.001 and tree complexity = 4. Variables were selected for each model using the "gbm.simplify" function. Model accuracy was good to excellent, based on the Area Under the receiver operating characteristic Curve (AUC = 0.82–0.93, Table S4). Model AUC was not noticeably affected by sample size (i.e., overall presence or sapling recruitment) among species.

As observed in previous work (Harris et al., 2022, 2024b), the strongest influence on sapling recruitment most species was the abundance of seedlings ≥ 1.5 m tall (Table S3). Most species also displayed negative relationships with live tree basal area (BA) and/or density, which speaks to the importance of light availability and canopy gaps in enabling sapling recruitment. While four of the models contained just seedling abundance and tree BA or density variables, the

other eight displayed other influences including understory vegetation cover, terrain, climate and soil characteristics (Table S3).

Calculating predicted recruitment density

Sapling recruitment models were used to generate the predicted probability of recruitment by RI subplot and species from both Time 1 (prior) and Time 2 (most recent) measurements. These probabilities were multiplied by the mean density of sapling recruits for each species when present to generate predicted sapling recruitment density. A comparison of mean predicted recruitment density for Time 2 with observed recruitment (Table S4) suggested that this method produced mean recruitment densities that were reasonably close to observed recruitment for most species. However, notable deviations from observed recruitment were seen in *Acer rubrum* (overprediction) and *Picea rubens* (underprediction). Although the sapling recruitment models all had good to excellent accuracy (Table S3), estimates of recruitment density could deviate from observed density if models are persistently over- or underestimating recruitment probability.

Appendix S2: Table S1. Variables considered in models of sapling recruitment.

Variable(s)	Source and details*
Conspecific and heterospecific seedling abundance	FIA: Seedling regeneration; Abundance within each of 6 Regeneration Indicator seedling height classes
Elevation	30-m Digital Elevation Model (DEM)
Slope steepness and aspect	FIA: Subplot
Live tree density, basal area, large tree density	FIA: Tree; large trees are ≥25.4 cm Diameter at Breast Height
Conspecific tree basal area	FIA: Tree
Forb, grass and shrub cover	FIA: Phase 2 vegetation subplot structure
Litter depth and duff depth	FIA: Condition down woody material calculation
Coarse woody material volume	FIA: Condition down woody material calculation
Topographic position index	Difference between mean elevation in 500-m window and elevation of focal pixel
Topographic roughness	Standard deviation of elevation in 500-m window
Topographic Wetness Index	Calculated via "r.topidx" function in GRASS GIS (Neteler et al., 2012)
Soil Organic Carbon	FIA: Condition (Domke et al., 2017)
Sand, silt and clay content	SoilGrids 2.0 (Poggio et al., 2021)
Soil pH and nitrogen content	SoilGrids 2.0 (Poggio et al., 2021)
Precipitation, temperature and maximum vapor pressure deficit	PRISM (Daly et al., 2008)
Percentage tree basal area mortality and percentage harvested	FIA: Tree; determined using cause of death codes
Mean December-March snow depth	National Snow and Ice Data Center (National Operational Hydrologic Remote Sensing Center, 2004)
Physiographic class	FIA: Condition

*Table names are given for variables derived from Forest Inventory and Analysis data.

Appendix S2: Table S2. Species used in sapling recruitment models with number of subplots (plots) in which each species was present and which had sapling recruitment at Time 2, along with mean (standard deviation) density of sapling recruits where present.

Species	Present	Sapling recruitment	Recruitment density (stems ha^{-1})
Abies balsamea	1026 (386)	119 (95)	1320 (1088)
Acer pensylvanicum	714 (337)	55 (49)	1051 (737)
Acer rubrum	1476 (611)	63 (55)	1423 (990)
Acer saccharum	784 (357)	21 (18)	1023 (496)
Betula alleghaniensis	544 (296)	42 (33)	1288 (1061)
Fagus grandifolia	824 (369)	113 (85)	1200 (1059)
Fraxinus americana	582 (288)	25 (22)	1037 (428)
Picea rubens	551 (272)	70 (58)	1175 (832)
Pinus strobus	332 (180)	18 (14)	823 (240)
Thuja occidentalis	265 (124)	25 (20)	1037 (524)
Tsuga canadensis	307 (161)	16 (14)	1065 (539)
All other species	1716 (715)	125 (99)	1251 (984)

Appendix S2: Table S3. Summary of sapling recruitment models including Area Under the receiver operating characteristic Curve (mean and standard deviation among folds) and relative influence of variables selected for each model.

Species	AUC	Variables
Abies balsamea	0.88 (0.02)	1.5–3.0 m tall (68%), total basal area (BA, 32%)
Acer pensylvanicum	0.86 (0.03)	>3.0 m tall (32%), total BA (22%), 1.5–3.0 m tall (17%), 30–90 cm tall heterospecific (16%), aspect (14%)
Acer rubrum	0.87 (0.03)	1.5–3.0 m tall (64%), total BA (36%)
Acer saccharum	0.93 (0.03)	>3.0 m tall (35%), snow depth (18%), May–October vapor pressure deficit (VPD, 17%), soil organic carbon (16%), forb cover (14%)
Betula alleghaniensis	0.93 (0.02)	1.5–3.0 m tall (32%), total BA (15%), aspect (14%), snow depth (14%), topographic position (10%), coarse woody material (10%), >3.0 m tall (7%)
Fagus grandifolia	0.84 (0.02)	1.5–3.0 m tall (23%), total BA (11%), >3.0 m tall (10%), total density (7%), conspecific BA (6%), November–April mean temperature (5%), November–April precipitation (5%), shrub cover (5%), soil organic carbon (5%), topographic roughness (5%), May–October precipitation (4%), topographic position (4%), May–October VPD (4%), topographic wetness index (4%), silt content (3%)
Fraxinus americana	0.84 (0.04)	total BA (40%), May–October mean temperature (30%), >3.0 m tall (30%)
Picea rubens	0.88 (0.02)	1.5–3.0 m tall (59%), total BA (41%)
Pinus strobus	0.93 (0.01)	1.5–3.0 m tall (61%), 91–152 cm tall heterospecific (39%)
Thuja occidentalis	0.88 (0.04)	1.5–3.0 m tall (65%), grass cover (35%)
Tsuga canadensis	0.92 (0.02)	1.5–3.0 m tall (35%), 30–91 cm tall heterospecific (34%), November–April VPD (32%)
All other species	0.9 (0.01)	>3.0 m tall (28%), 1.5–3.0 m tall (15%), total BA (11%), topographic roughness (9%), soil organic carbon (8%), May–October precipitation (8%), topographic wetness index (8%), total density (7%), 91–152 cm tall (6%)

Appendix S2: Table S4. Mean (standard deviation) of sapling recruitment (stems ha^{-1} $year^{-1}$) for the 20 most abundant tree species in the most recent measurement period (2018–2023) as observed in all FIA subplots and Regeneration Indicator subplots, and as predicted by sapling recruitment models using probability of recruitment and threshold values for presence/absence.

Species	All	Regeneration Indicator	Predicted
Abies balsamea	12.3 (74.9)	9.5 (60.7)	10.4 (30.8)
Fagus grandifolia	6.8 (42.3)	8.0 (51.7)	8.3 (27.9)
Acer rubrum	6.0 (51)	5.8 (44.1)	10.0 (31.2)
Picea rubens	5.8 (45)	5.2 (39.7)	2.9 (10.6)
Acer pensylvanicum	3.1 (29.5)	3.5 (31.7)	4.6 (16.1)
Betula alleghaniensis	2.5 (29.1)	3.3 (33)	3.2 (14.4)
Thuja occidentalis	1.9 (27.7)	1.6 (18.8)	2.0 (12.1)
Tsuga canadensis	1.6 (20.1)	1.1 (15.8)	1.0 (8.8)
Acer saccharum	1.5 (19.6)	1.4 (17.2)	2.5 (10.8)
Populus tremuloides	1.5 (33.1)	2.1 (46.9)	2.3 (20.4)
Pinus strobus	1.4 (18.8)	0.9 (11.6)	0.1 (0.6)
Fraxinus americana	1.4 (18.2)	1.5 (17.4)	1.5 (6.8)
Betula papyrifera	1.1 (19.5)	0.7 (11.9)	1.0 (6.6)
Betula populifolia	0.8 (20)	0.0 (2.7)	0.1 (1.7)
Picea mariana	0.7 (14.1)	0.3 (6.6)	0.3 (3.5)
Betula lenta	0.6 (14)	0.3 (6.2)	0.2 (2.4)
Ostrya virginiana	0.6 (11.5)	1.4 (22.2)	1.2 (9.9)
Picea glauca	0.5 (12.1)	0.1 (3.9)	0.2 (1.3)
Prunus pensylvanica	0.5 (15.1)	0.6 (15.2)	1.1 (10.8)
Quercus rubra	0.5 (12.7)	0.8 (13.9)	0.7 (5.3)

References

Daly, C., Halbleib, M., Smith, J. I., Gibson, W. P., Doggett, M. K., Taylor, G. H., Curtis, J., & Pasteris, P. P. (2008). Physiographically sensitive mapping of climatological temperature and precipitation across the conterminous United States. *International Journal of Climatology*, *28*, 2031–2064. https://doi.org/10.1002/joc

Domke, G. M., Perry, C. H., Walters, B. F., Nave, L. E., Woodall, C. W., & Swanston, C. W. (2017). Toward inventory-based estimates of soil organic carbon in forests of the United States. *Ecological Applications*, *27*(4), 1223–1235. https://doi.org/10.1002/eap.1516

Elith, J., Leathwick, J. R., & Hastie, T. (2008). A working guide to boosted regression trees. *The Journal of Animal Ecology*, *77*(4), 802–813. https://doi.org/10.1111/j.1365-2656.2008.01390.x

Harris, L. B., Woodall, C. W., & D'Amato, A. W. (2022). Increasing the utility of tree regeneration inventories: Linking seedling abundance to sapling recruitment. *Ecological Indicators*, *145*, 109654. https://doi.org/10.1016/j.ecolind.2022.109654

Harris, L. B., Woodall, C. W., & D'Amato, A. W. (2024a). Relationships between juvenile tree survival and tree density, shrub cover and temperature vary by size class based on ratios of abundance. *Canadian Journal of Forest Research*, *54*, 122–133. https://doi.org/10.1139/cjfr-2023-0097

Harris, L. B., Woodall, C. W., & D'Amato, A. W. (2024b). Sapling recruitment as an indicator of carbon resiliency in forests of the northern USA. *Ecology and Evolution*, *14*, e70077. https://doi.org/10.1002/ece3.70077

Hijmans, R. J., Phillips, S., R. Leathwick, J., & Elith, J. (2021). *dismo: Species Distribution Modeling* (1.3-5). https://doi.org/https://cran.r-project.org/web/packages/dismo/index.html

National Operational Hydrologic Remote Sensing Center. (2004). *Snow Data Assimilation System (SNODAS) Data Products at NSIDC, Version 1*. National Snow and Ice Data Center.

Poggio, L., De Sousa, L. M., Batjes, N. H., Heuvelink, G. B. M., Kempen, B., Ribeiro, E., & Rossiter, D. (2021). SoilGrids 2.0: Producing soil information for the globe with quantified spatial uncertainty. *SOIL*, *7*(1), 217–240. https://doi.org/10.5194/soil-7-217-2021
